# Complete two-loop QCD contributions to the lightest Higgs-boson mass in the MSSM with complex parameters

**DOI:** 10.1140/epjc/s10052-018-6055-y

**Published:** 2018-07-14

**Authors:** Sophia Borowka, Sebastian Paßehr, Georg Weiglein

**Affiliations:** 1 Theoretical Physics Department, CERN, Geneva, Switzerland; 2 Laboratoire de Physique Théorique et Hautes Énergies (LPTHE), CNRS, Sorbonne Université, 4 Place Jussieu, 75252 Paris Cedex 05, France; 3 Deutsches Elektronensynchrotron DESY, Notkestraße 85, 22607 Hamburg, Germany

## Abstract

Higher-order corrections to the MSSM Higgs-boson masses are desirable for accurate predictions currently testable at the LHC. By comparing the prediction with the measured value of the discovered Higgs signal, viable parameter regions can be inferred. For an improved theory accuracy, we compute all two-loop corrections involving the strong coupling for the Higgs-boson mass spectrum of the MSSM with complex parameters. Apart from the dependence on the strong coupling, these contributions depend on the weak coupling and Yukawa couplings, leading to terms of $\mathcal {O}{\left( \alpha \alpha _s\right) }$ and $\mathcal {O}{\left( \sqrt{\alpha _{q_1}}\sqrt{\alpha _{q_2}}\alpha _s\right) }$, ($q_{1,2}=t,b,c,s,u,d$). The full dependence on the external momentum and all relevant mass scales is taken into account. The calculation is performed in the Feynman-diagrammatic approach which is flexible in the choice of the employed renormalization scheme. For the phenomenological results presented here, a renormalization scheme consistent with higher-order corrections included in the code FeynHiggs is adopted. For the evaluation of the results, a total of 513 two-loop two-point integrals with up to five different mass scales are computed fully numerically using the program SecDec. A comparison with existing results in the limit of real parameters and/or vanishing external momentum is carried out, and the impact on the lightest Higgs-boson mass is discussed, including the dependence on complex phases. The new results will be included in the public code FeynHiggs.

## Introduction

Since the discovery of a signal in the Higgs-boson searches at the LHC [1, 2] with a mass around 125 GeV, it is a prime goal to reveal the detailed nature of the new particle. While with the present experimental and theoretical uncertainties the measured properties of the detected particle are compatible with the expectations for the Higgs boson of the Standard Model (SM) [3, 4], other interpretations corresponding to very different underlying physics are also in agreement with the data. A crucial question in this context is in particular whether the observed particle is part of an extended Higgs sector that would be associated with a more general theoretical framework beyond the SM.

Within the theoretically well motivated Minimal Supersymmetric extension of the SM (MSSM), the observed particle can be interpreted as a light state within a richer spectrum of scalar particles.^1^ The Higgs-boson sector of the MSSM consists of two complex scalar doublets leading to five physical Higgs bosons and three (would-be) Goldstone bosons. At the tree-level, the physical states are given by the neutral *CP*-even bosons *h*, *H* and the *CP*-odd state *A*, together with the charged $H^{\pm }$ bosons. The Higgs sector at lowest order can be parametrized in terms of the *A*-boson mass $m_A$ and the ratio of the vacuum expectation values of the scalar doublets, $\tan \beta = \left. v_2\big /v_1\right.$. The MSSM with complex parameters (cMSSM) is of particular interest since it provides new sources of *CP*-violation in addition to the *CP*-violating phase of the SM. Thereby the Higgs sector is *CP*-conserving at the tree level, but potentially large loop contributions involving complex parameters from other supersymmetric (SUSY) sectors can lead to an admixture of the *CP*-even states $h,\,H,$ and the *CP*-odd *A* resulting in the mass eigenstates $h_1, h_2, h_3$ [6–10]. In this case $m_A$ is no longer a useful input parameter; instead the mass of the charged Higgs boson $m_{H^\pm }$ is used. Besides the input parameter $m_A$ or $m_{H^\pm }$ all other Higgs-boson masses are predicted quantities in the MSSM. The Higgs-boson masses and mixings in the neutral sector are strongly affected by loop contributions. Especially for the experimentally measured Higgs boson at about 125 GeV a sufficiently high accuracy of the theoretical computation is essential for drawing reliable conclusions on the viability of the investigated region of parameter space.

A large amount of work has been invested into calculating higher-order corrections to the mass spectrum within the MSSM with real parameters [11–67] as well as the MSSM with complex parameters [6–10, 65–76]. The largest loop contributions originate from the Yukawa sector due to the size of the top-quark Yukawa coupling $h_t$, where $\alpha _t=\left. h_t^2\big /(4\pi )\right.$. At the two-loop level QCD corrections enter. The dominant contribution at the two-loop level is given by the $\mathcal {O}{\left( \alpha _{t}\alpha _{s}\right) }$ terms which are known for the MSSM with complex parameters [12–14, 72]. Also the $\mathcal {O}{\left( \alpha _{t}^2+\alpha _t\alpha _b+\alpha _b^2\right) }$ corrections are known for the case of complex parameters [73–75]. Restricting to the case of real parameters, the momentum-dependent $\mathcal {O}{\left( \alpha _{t}\alpha _{s}\right) }$ corrections [53–55] and the contributions for the case where all Yukawa couplings except the one of the top quark are neglected [54] are known. While the phases of the complex parameters affect the predictions for the Higgs-boson masses, production cross sections [77] and decays [78–80], they also induce *CP*-violating effects that are constrained by other experiments. These concern in particular the electric dipole moments [81–88]. For the usual convention where the phase of the mass of the electroweakinos, $\phi _{M_2}$, is set to zero without loss of generality, the phase of the parameter $\mu$ is constrained to be very close to zero or $\pi$. The other important phases of the gluino mass, $\phi _{M_3}$, and the trilinear soft-breaking parameters of the stops, $\phi _{A_t}$, and sbottoms, $\phi _{A_b}$, are much less constrained. In particular, the bounds on the phases of the trilinear soft-breaking parameters are significantly weaker for the third generation than for the second and first generation.

In this article the full two-loop QCD corrections to the Higgs-boson masses are presented for the general case of the MSSM with complex parameters. They contain all previously computed results for the MSSM with real or complex parameters. The contributions are comprised of the $\mathcal {O}{\left( \alpha \alpha _s\right) }$ terms, involving the electroweak gauge coupling $\alpha$, and the $\mathcal {O}{\left( \sqrt{\alpha _{q_1}}\sqrt{\alpha _{q_2}}\alpha _s\right) }$ terms, involving the Yukawa couplings $\alpha _{q_1}$, $\alpha _{q_2}$, where $q_{1,2}=t,b,c,s,u,d$. Terms with mixed up- and down-type Yukawa couplings only appear in conjunction with $m_{H^\pm }$ as input parameter. Mixed contributions of $\mathcal {O}{\left( \sqrt{\alpha }\sqrt{\alpha _{q_1}}\alpha _s\right) }$ involving one gauge coupling and one Yukawa coupling do not appear in the final result. The results obtained here for the MSSM can furthermore be used as an approximation for higher-order contributions within the NMSSM, as discussed in Refs. [89–91]. The computation carried out below makes use of previously developed tools [53, 92, 93]. The momentum-dependent two-loop integrals appearing in the two-loop QCD corrections are evaluated with an adapted version of SecDec 2 [94–96]. For the numerical analysis the new contributions are combined with the full one-loop result [10] and the leading $\mathcal {O}{\left( \alpha _{t}^2\right) }$ terms [73, 74] in the Feynman-diagrammatic approach for complex parameters, available through the public program FeynHiggs [10–12, 78, 97]. In deriving the new contributions the renormalization scheme of Ref. [10] at the one-loop level has been adopted and applied to the case of the $\mathcal {O}{\left( \alpha \alpha _s\right) }$ contributions. This ensures that the obtained analytical results for the renormalized two-loop self-energies can consistently be incorporated into FeynHiggs. In the results presented in this paper no resummation of higher-order logarithmic contributions as obtained in Refs. [56–62] has been included. The combination of resummed higher-order logarithmic contributions with the results obtained in the present paper will be addressed in future work. In the numerical analysis below, we show results for the masses of the three neutral Higgs bosons of the MSSM with complex parameters and their phase dependence, with a particular focus on those results which are phenomenologically most relevant.

The paper is organized as follows: Sect. 2 provides the theoretical framework for the calculation and renormalization of the Feynman diagrams that is used to arrive at expressions for the dressed propagators of the Higgs sector up to the two-loop level. The calculation of the unrenormalized self-energies and the construction of the two-loop counterterms are described in Sect. 3. Details on the numerical evaluation of the momentum-dependent two-loop integrals are given in Sect. 4, whereas the impact of the new contributions on the Higgs-boson masses is discussed in Sect. 5. The conclusions are given in Sect. 6.

## The Higgs sector of the MSSM with complex parameters

### Tree-level relations for masses and mixing

The two scalar *SU*(2)-doublets are conventionally expressed in terms of their components as follows,

2.1 $$\begin{aligned} \begin{aligned} \mathcal {H}_{1}&= \begin{pmatrix} v_{1} + \frac{1}{\sqrt{2}}(\phi _{1} - {i}\chi _{1})\\ -\phi ^{-}_{1}\end{pmatrix},\\ \mathcal {H}_{2}&= \mathrm {e}^{{i}\xi }\begin{pmatrix} \phi ^{+}_{2}\\ v_{2} + \frac{1}{\sqrt{2}}(\phi _{2} + {i}\chi _{2})\end{pmatrix}, \end{aligned} \end{aligned}$$

with the relative phase $\xi$. The Higgs potential can be written as a polynomial in the field components,

2.2 $$\begin{aligned} V_{H}&= -T_{\phi _{1}} \phi _{1} - T_{\phi _{2}} \phi _{2} - T_{\chi _{1}} \chi _{1} - T_{\chi _{2}} \chi _{2}\nonumber \\&\quad + \frac{1}{2}\begin{pmatrix} \phi _{1},&\phi _{2},&\chi _{1},&\chi _{2} \end{pmatrix} \begin{pmatrix}\mathbf {M}_{\phi } &{} \mathbf {M}_{\phi \chi }\\ \mathbf {M}_{\phi \chi }^{\dagger } &{} \mathbf {M}_{\chi } \end{pmatrix} \begin{pmatrix} \phi _{1},&\phi _{2},&\chi _{1},&\chi _{2}\end{pmatrix}^{\text {T}}\nonumber \\&\quad + \begin{pmatrix} \phi ^{-}_{1},&\phi ^{-}_{2}\end{pmatrix} \mathbf {M}_{\phi ^{\pm }} \begin{pmatrix} \phi ^{+}_{1}\\ \phi ^{+}_{2}\end{pmatrix} + \cdots , \end{aligned}$$

where terms of third and fourth power in the fields have been omitted, and the relations $\phi ^{-}_{1} = \left( \phi ^{+}_{1}\right) ^{\dagger }$ and $\phi ^{-}_{2} = \left( \phi ^{+}_{2}\right) ^{\dagger }$ have been used. Explicit expressions for the tadpole coefficients *T* and for the mass matrices $\mathbf {M}$ can be found in Ref. [10]. They are parametrized by the phase $\xi$, the real SUSY-breaking quantities $m_{1,2}^{2} = \tilde{m}_{1,2}^{2} + |\mu |^{2}$, and the complex SUSY-breaking quantity $m_{12}^{2}$. With the help of a Peccei–Quinn transformation [98] the parameter $m_{12}^{2}$ can be redefined such that its phase vanishes [99], leaving only the phase $\xi$ as a potential source of *CP*-violation at tree level. The requirement of minimizing $V_{H}$ at the vacuum expectation values $v_{1}$ and $v_{2}$ is equivalent to the requirement of vanishing tadpoles of the physical fields, which in turn implies the condition $\xi = 0$ at tree level. As a consequence, the Higgs sector of the MSSM is *CP*-conserving at lowest order. This implies in Eq. (2.2) that $\mathbf {M}_{\phi \chi }$ is equal to zero, and $\phi _{1,2}$ do not mix with $\chi _{1,2}$ at tree-level.

The remaining $(2\times 2)$-matrices $\mathbf {M}_{\phi }$, $\mathbf {M}_{\chi }$, $\mathbf {M}_{\phi ^{\pm }}$ can be transformed into the mass eigenstate basis with the help of orthogonal matrices *D*(*x*), using the abbreviations $s_{x} \equiv \sin {x}$, $c_{x} \equiv \cos {x}$,

2.3 $$\begin{aligned} \begin{aligned} D{\left( x\right) }&= \begin{pmatrix}-s_{x} &{} c_{x}\\ c_{x} &{} s_{x}\end{pmatrix},&\quad \begin{pmatrix} h\\ H \end{pmatrix}&= D(\alpha ) \begin{pmatrix} \phi _{1}\\ \phi _{2}\end{pmatrix},\\ \begin{pmatrix} A\\ G \end{pmatrix}&= D(\beta _{n}) \begin{pmatrix} \chi _{1}\\ \chi _{2}\end{pmatrix},&\quad \begin{pmatrix} H^{\pm }\\ G^{\pm } \end{pmatrix}&= D(\beta _{c}) \begin{pmatrix} \phi ^{\pm }_{1}\\ \phi ^{\pm }_{2}\end{pmatrix}. \end{aligned} \end{aligned}$$

The Higgs potential in this basis can be expressed as follows,

2.4 $$\begin{aligned} V_{H}&= -T_h \, h- T_H \, H - T_A \, A - T_G\, G\nonumber \\&\quad + \frac{1}{2}\begin{pmatrix} h,&H,&A,&G \end{pmatrix} \mathbf {M}_{hHAG} \begin{pmatrix} h,&H,&A,&G \end{pmatrix}^{\text {T}}\nonumber \\&\quad + \begin{pmatrix} H^{-},&G^{-}\end{pmatrix} \mathbf {M}_{H^\pm G^\pm } \begin{pmatrix} H^{+}\\ G^{+}\end{pmatrix} + \cdots \end{aligned}$$

with the tadpole coefficients $T_{h,H,A,G}$ and the mass matrices

2.5 $$\begin{aligned} \mathbf {M}_{hHAG}&= \begin{pmatrix} m^2_{h} &{} m^2_{hH} &{} m^2_{hA} &{} m^2_{hG} \\ m^2_{hH} &{} m^2_{H} &{} m^2_{HA} &{} m^2_{HG} \\ m^2_{hA} &{} m^2_{HA} &{} m^2_{A} &{} m^2_{AG} \\ m^2_{hG} &{} m^2_{HG} &{} m^2_{AG} &{} m^2_{G} \end{pmatrix} , \nonumber \\ \mathbf {M}_{H^\pm G\pm }&= \begin{pmatrix} m^2_{H^\pm } &{} m^2_{H^-G^+} \\ m^2_{G^-H^+} &{} m^2_{G^\pm } \end{pmatrix} ; \end{aligned}$$

explicit expressions for the entries are given in Ref. [10]. The tadpole terms in Eq. (2.4) are zero at the tree level, but they enter the predictions for the Higgs-boson masses at higher orders. As mentioned above, the ellipses denote terms of higher power in the fields which are not relevant in our calculation.

After applying the minimization conditions to Eq. (2.5), the mass matrices can be brought into canonical form^2^

2.6 $$\begin{aligned} \mathbf {M}_{ h H A G}^{(0)}&= {\mathrm {diag}} \left( m_h^2,\, m_H^2,\, m_A^2,\, m_G^2 \right) ,\nonumber \\ \mathbf {M}_{H^\pm G^\pm }^{(0)}&= {\mathrm {diag}} \left( m_{H^\pm }^2,\, m_{G^\pm }^2 \right) , \end{aligned}$$

for $\beta = \beta _{n} = \beta _{c}$, with $\beta \in \left[ 0,\pi /2\right)$ given in terms of the vacuum expectation values,

2.7 $$\begin{aligned} \tan \beta&\equiv t_\beta = \frac{v_2}{v_1}, \end{aligned}$$

and for the second mixing angle $\alpha \in \left[ -\pi /2,0\right)$ determined by

2.8 $$\begin{aligned} \tan (2\alpha )&= \frac{m_{A}^2 + m_Z^2}{m_A^2 - m_Z^2}\, \tan (2\beta ). \end{aligned}$$

The Goldstone bosons are massless,^3^ $m_{G^\pm } = m_G = 0$. The masses $m_{H^\pm }, m_A, m_h, m_H$ fulfil the relations

2.9a $$\begin{aligned} m_{H^{\pm }}^{2}&= m_{A}^{2} + M_{W}^{2}, \end{aligned}$$

2.9b $$\begin{aligned} m_{h,\,H}^2&= \frac{1}{2}\left( m_{A}^{2} + M_{Z}^{2}\mp \sqrt{\left( m_{A}^{2} + M_{Z}^{2}\right) ^{2} - 4 m_{A}^{2} M_{Z}^{2}\, c_{2\beta }^{2}}\right) , \end{aligned}$$

including the vector-boson masses $M_W$ and $M_Z$. Given the relation in Eq. (2.9a), both $m_A$ and $m_{H^{\pm }}$ can be chosen as input parameter.

At lowest order, the irreducible two-point vertex functions of the neutral Higgs sector

2.10 $$\begin{aligned} \Gamma ^{(0)}_{hHAG}(p^2)&= \, i \, \Big [ p^2 \mathbf 1 - \mathbf {M}^{(0)}_{hHAG} \Big ] \end{aligned}$$

are diagonal, and the entries of the mass matrices in Eq. (2.6) provide the poles of the diagonal lowest-order propagators

2.11 $$\begin{aligned} \Delta ^{(0)}_{hHAG} (p^2) =\, - \Big [ \Gamma ^{(0)}_{hHAG}(p^2) \Big ]^{-1}. \end{aligned}$$

### Masses and mixing beyond lowest order

Going beyond leading order, the irreducible two-point functions are dressed by adding the matrix $\hat{\varvec{\Sigma }}_{h H A G}$ of the renormalized diagonal and non-diagonal self-energies for the *h*, *H*, *A*, *G* fields up to the considered order,

2.12 $$\begin{aligned} p^2 \mathbf 1 - \mathbf {M}^{(0)}_{hHAG}&\rightarrow p^2 \mathbf 1 - \mathbf {M}^{(0)}_{hHAG} +\hat{\varvec{\Sigma }}_{hHAG}(p^2) \nonumber \\&\equiv p^2 \mathbf 1 - \mathbf {M}_{hHAG}(p^2), \end{aligned}$$

yielding the full renormalized two-point vertex function

2.13 $$\begin{aligned} \hat{\Gamma }_{hHAG}(p^2) = \, i \, \Big [ p^2 \mathbf 1 - \mathbf {M}_{hHAG} \Big ]\;. \end{aligned}$$

The latter generally contains a mixing of all fields with equal quantum numbers. The dressed propagators are obtained by inverting the matrix $\hat{\Gamma }_{hHAG}(p^2)$.

Truncating the perturbative expansion at the two-loop level, the momentum-dependent corrections to the neutral Higgs-boson mass matrices in Eq. (2.12) are given by

2.14 $$\begin{aligned} \mathbf {M}_{ h H A G}^{(2)} (p^2) = \mathbf {M}_{ h H A G}^{(0)} - \hat{\varvec{\Sigma }}_{h H A G}^{(1)}(p^2) - \hat{\varvec{\Sigma }}_{h H A G}^{(2)}(p^2) \ . \end{aligned}$$

For the MSSM with complex parameters, the one-loop self-energies are completely known [10], and the leading two-loop $\mathcal {O}{\left( \alpha _t \alpha _s\right) }$ and $\mathcal {O}{\left( \alpha _{t}^2+\alpha _t\alpha _b+\alpha _b^2\right) }$ contributions have been obtained in the approximation of zero external momentum [72–75]. In the case of the MSSM with real parameters also the momentum-dependent corrections of $\mathcal {O}{\left( \alpha _{t}\alpha _{s}\right) }$ are known [53–55]. The remaining QCD contributions at the two-loop level are completed within this paper. These contributions comprise terms of the $\mathcal {O}{\left( \alpha _x \alpha _s\right) }$, where $\alpha _x$ is either the gauge coupling $\alpha$ or the Yukawa coupling $\alpha _q$ with $q = \{u,d,s,c,b,t\}$. We neglect CKM mixing for those contributions.

In order to obtain the physical Higgs-boson masses from the dressed propagators at the considered order, it is sufficient to explicitly derive the entries of the $(3\times 3)$-submatrix of Eq. (2.14) corresponding to the (*h*, *H*, *A*)-components. A mixing of the neutral Higgs bosons with the Goldstone boson, as well as Goldstone-*Z* mixing, yields subleading two-loop contributions to the Higgs-boson masses that are not of $\mathcal {O}{\left( \alpha _x \alpha _s\right) }$.

The masses of the three neutral Higgs bosons are obtained from the real parts of the complex poles of the (*h*, *H*, *A*)-propagator matrix. They are obtained as the zeroes of the determinant of the renormalized two-loop two-point vertex function,^4^

2.15 $$\begin{aligned}&\left. {\text {det}}\hat{\Gamma }^{(2)}_{hHA}{\left( p^2\right) }\right| _{p^2\,=\,M_j^2\,-\,{i}\,M_j\,\Gamma _j} = 0, \nonumber \\&\hat{\Gamma }^{(2)}_{hHA}{\left( p^2\right) } = {i}\left[ p^2 \mathbf{1 } - \mathbf {M}_{hHA}^{(2)}{\left( p^2\right) }\right] ,\quad j \in \{h,H,A\},\nonumber \\ \end{aligned}$$

with $\mathbf {M}_{hHA}^{(2)}$ being the corresponding $(3\times 3)$-submatrix of Eq. (2.14). The impact of the self-energies on the mixing and couplings of the various Higgs bosons to other (MS)SM particles can be obtained with the same formalism as described in Refs. [10, 100].

## Calculation of the renormalized two-loop self-energies

The renormalized two-loop self-energies can be written as

3.1 $$\begin{aligned} \hat{\varvec{\Sigma }}_{hHA} ^{(2)} (p^2) = \varvec{\Sigma }_{hHA} ^{(2)} (p^2) - \delta ^{(2)} \mathbf {M}^{\mathbf {Z}}_{hHA}, \end{aligned}$$

where $\varvec{\Sigma }_{hHA} ^{(2)}$ denotes the unrenormalized self-energies corresponding to the sum of genuine two-loop diagrams and one-loop diagrams with counterterm insertions. The symbol $\delta ^{(2)}\mathbf {M}^{\mathbf {Z}}_{hHA}$ comprises all two-loop counterterms resulting from parameter and field renormalization.

**Fig. 1:**
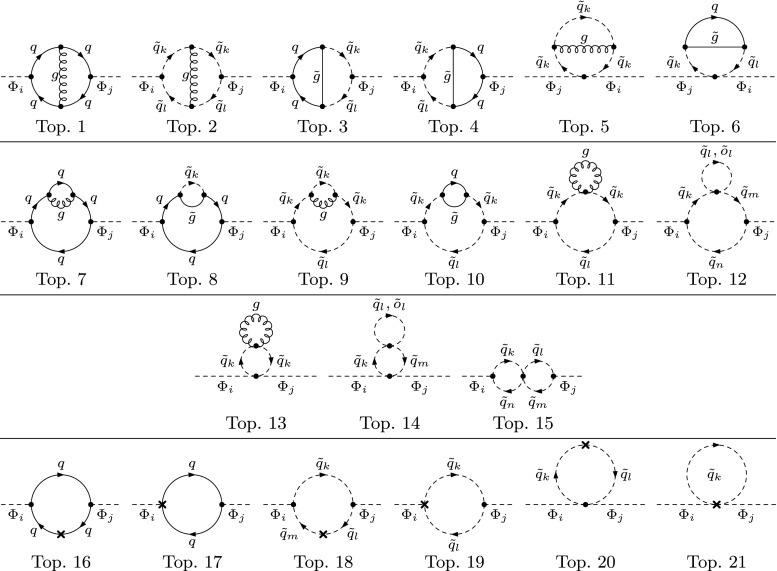
Types of two-loop self-energy diagrams for the neutral Higgs bosons. One-loop counterterm insertions are denoted by a cross. $\Phi _{i} = h,\,H,\,A$; $\;\tilde{q} \ne \tilde{o}$. Topologies 11 and 13 contain a one-point loop with a mass-less gluon and are therefore equal to zero

The contributing types of Feynman diagrams for the calculation of the full two-loop QCD corrections entering Eq. (3.1) are depicted in Fig. 1. The diagrams of the topologies 12, 14 and 15 contribute only if all squarks have the same flavor; couplings with different flavors vanish since the color sum is equal to zero in that case. The diagrammatic calculation has been performed with the help of FeynArts [101, 102] in generating the Feynman diagrams, and TwoCalc [92] and Reduze [103] for the two-loop trace evaluation and tensor reduction. The one-loop renormalization constants have been computed with the help of FormCalc [104].

### Two-loop counterterms

In order to obtain the renormalized self-energies in Eq. (3.1), counterterms have to be introduced up to second order in the loop expansion, for the tadpoles

3.2 $$\begin{aligned} T_i \rightarrow T_i + \delta ^{(1)}T_i + \delta ^{(2)} T_i \, , \quad i=h,\,H,\,A, \end{aligned}$$

and for the mass matrices of Eq. (2.4)

3.3a $$\begin{aligned} \mathbf {M}_{ h H A}&\rightarrow \mathbf {M}_{ h H A}^{(0)} + \delta ^{(1)} \mathbf {M}_{ h H A} + \delta ^{(2)} \mathbf {M}_{ h H A}\ , \end{aligned}$$

3.3b $$\begin{aligned} \delta ^{(k)}\mathbf {M}_{hHA}&= \begin{pmatrix}\delta ^{(k)}m_{h}^{2} &{} \delta ^{(k)}m_{hH}^{2} &{} \delta ^{(k)}m_{hA}^{2} \\ \delta ^{(k)}m_{Hh}^{2} &{} \delta ^{(k)}m_{H}^{2} &{} \delta ^{(k)}m_{HA}^{2}\\ \delta ^{(k)}m_{Ah}^{2} &{} \delta ^{(k)}m_{AH}^{2} &{} \delta ^{(k)}m_{A}^{2}\end{pmatrix}, \end{aligned}$$

3.3c $$\begin{aligned} m^2_{H^\pm }&\rightarrow m_{H^\pm }^{2} +\, \delta ^{(1)} m^2_{H^\pm } +\, \delta ^{(2)} m^2_{H^\pm }. \end{aligned}$$

The two-loop counterterms of $\mathcal {O}{\left( \alpha \alpha _s\right) }$ have the same structure as the corresponding one-loop counterterms. They are listed here for completeness and to fix our notation.

In order to ensure the correct form of the counterterms for the mass matrices, the rotation angles $\beta _n$ and $\beta _c$ from Eq. (2.3) have to be distinguished from $\beta$ in Eq. (2.7). Whereas no renormalization is needed for $\alpha$, $\beta _n$ and $\beta _c$, a counterterm associated with $\beta$ of the form $\beta \rightarrow \beta +\delta \beta$ is required, in accordance with the renormalization of $\tan \beta$,

3.4 $$\begin{aligned} t_\beta \rightarrow t_\beta + \delta ^{(1)}t_\beta + \delta ^{(2)}t_\beta \ . \end{aligned}$$

In the resulting expressions for the counterterm matrices, the identification $\beta _c =\beta _n = \beta$ can be made, see Ref. [10] for details of the analogous treatment at the one-loop order (note that a different convention for the counterterm of $t_\beta$ is used in Ref. [10]). A complete list of the two-loop counterterms is given in the Appendix of Ref. [74].

In addition to the parameter renormalization described previously, the field-renormalization constants $\delta ^{(1)}Z_{\mathcal {H}_{i}}$ and $\delta ^{(2)}Z_{\mathcal {H}_{i}}$ are introduced at the one-loop and two-loop order (restricting the latter to the contributions of $\mathcal {O}{\left( \alpha \alpha _s\right) }$) for each of the scalar doublets of Eq. (2.1) through the transformation

3.5 $$\begin{aligned} \mathcal {H}_{i} \rightarrow \sqrt{Z_{\mathcal {H}_{i}}}\mathcal {H}_{i}&= \left[ 1 + \frac{1}{2}\delta ^{(1)}Z_{\mathcal {H}_{i}} + \frac{1}{2}\delta ^{(2)}Z_{\mathcal {H}_{i}} \right] \mathcal {H}_{i}. \end{aligned}$$

The field-renormalization constants in the mass-eigenstate basis of Eq. (2.3) are obtained via

3.6a $$\begin{aligned} \begin{pmatrix}h \\ H \end{pmatrix}&\rightarrow D(\alpha )\begin{pmatrix}\sqrt{Z_{\mathcal {H}_{1}}} &{} 0\\ 0 &{} \sqrt{Z_{\mathcal {H}_{2}}}\end{pmatrix} D(\alpha )^{-1} \begin{pmatrix} h \\ H \end{pmatrix}\nonumber \\&\equiv \mathbf {Z}_{hH} \begin{pmatrix}h\\ H \end{pmatrix}, \end{aligned}$$

3.6b $$\begin{aligned} \begin{pmatrix}A\\ G \end{pmatrix}&\rightarrow D(\beta _{n})\begin{pmatrix}\sqrt{Z_{\mathcal {H}_{1}}} &{} 0\\ 0 &{} \sqrt{Z_{\mathcal {H}_{2}}}\end{pmatrix} D(\beta _{n})^{-1} \begin{pmatrix}A\\ G \end{pmatrix}\nonumber \\&\equiv \begin{pmatrix}Z_{AA} &{} Z_{AG} \\ Z_{GA} &{} Z_{GG} \end{pmatrix} \begin{pmatrix}A\\ G\end{pmatrix} \; \equiv \; \mathbf {Z}_{AG} \begin{pmatrix}A\\ G\end{pmatrix}, \end{aligned}$$

3.6c $$\begin{aligned} \begin{pmatrix}H^{\pm }\\ G^{\pm }\end{pmatrix}&\rightarrow D(\beta _{c})\begin{pmatrix}\sqrt{Z_{\mathcal {H}_{1}}} &{} 0\\ 0 &{} \sqrt{Z_{\mathcal {H}_{2}}}\end{pmatrix} D(\beta _{c})^{-1} \begin{pmatrix}H^{\pm }\\ G^{\pm }\end{pmatrix}\nonumber \\&\equiv \mathbf {Z}_{H^{\pm }G^{\pm }} \begin{pmatrix}H^{\pm }\\ G^{\pm } \end{pmatrix} . \end{aligned}$$

The matrices $\mathbf {Z}_{ij}$ can be expanded as

3.7 $$\begin{aligned} \mathbf {Z}_{ij} = \mathbf {1} + \delta ^{(1)}\mathbf {Z}_{ij} + \delta ^{(2)}\mathbf {Z}_{ij}\, . \end{aligned}$$

The required one-loop expressions for the entries in the $\delta ^{(1)}\mathbf {Z}_{ij}$-matrices are given in Ref. [10]; the corresponding set of two-loop expressions is given in Ref. [74].

The genuine two-loop counterterms $\delta ^{(2)} \mathbf {M}^{\mathbf {Z}}_{hHA}$ of Eq. (3.1) can now be summarized as

3.8 $$\begin{aligned} \delta ^{(2)}\mathbf {M}^{\mathbf {Z}}_{hHA}&= \delta ^{(2)}\mathbf {M}_{hHA} + \begin{pmatrix} \delta ^{(2)}\mathbf {Z}_{hH}^T &{} \mathbf {0} \\ \mathbf {0} &{} \delta ^{(2)}Z_{AA} \end{pmatrix}\nonumber \\&\quad \times \left( \mathbf {M}_{hHA}^{(0)} - p^2 \mathbf 1 \right) \nonumber \\&\quad + \left( \mathbf {M}_{hHA}^{(0)} - p^2 \mathbf 1 \right) \begin{pmatrix} \delta ^{(2)}\mathbf {Z}_{hH} &{} \mathbf {0} \\ \mathbf {0} &{} \delta ^{(2)}Z_{AA} \end{pmatrix}, \end{aligned}$$

where the required two-loop mass counterterms read

3.9a $$\begin{aligned} \delta ^{(2)}m_{h}^{2}&= c_{\alpha -\beta }^{2}\,\delta ^{(2)}m_A^{2} + s_{\alpha +\beta }^{2}\,\delta ^{(2)}m_Z^{2} \nonumber \\&\quad + c_{\beta }^{2}\,\delta ^{(2)}t_{\beta } \left( s_{2(\alpha -\beta )}\,m_A^{2} + s_{2(\alpha +\beta )}\,m_Z^{2}\right) + \frac{e\,s_{\alpha -\beta }}{2\,M_{W}\,s_{\text {w}}} \nonumber \\&\quad \times \Biggl [\left( 1 + c_{\alpha -\beta }^{2}\right) \delta ^{(2)}T_{h} + s_{\alpha -\beta }\,c_{\alpha -\beta }\,\delta ^{(2)}T_{H}\Biggr ], \end{aligned}$$

3.9b $$\begin{aligned} \delta ^{(2)}m_{H}^{2}&= s_{\alpha -\beta }^{2}\,\delta ^{(2)}m_A^{2} + c_{\alpha +\beta }^{2}\,\delta ^{(2)}m_Z^{2} \nonumber \\&\quad - c_{\beta }^{2}\,\delta ^{(2)}t_{\beta } \left( s_{2(\alpha -\beta )}\,m_A^{2} + s_{2(\alpha +\beta )}\,m_Z^{2}\right) - \frac{e\,c_{\alpha -\beta }}{2\,M_{W}\,s_{\text {w}}} \nonumber \\&\quad \times \Biggl [\left( 1 + s_{\alpha -\beta }^{2}\right) \delta ^{(2)}T_{H} + c_{\alpha -\beta }\,s_{\alpha -\beta }\,\delta ^{(2)}T_{h}\Biggr ], \end{aligned}$$

3.9c $$\begin{aligned} \delta ^{(2)}m_{A}^{2}&= \delta ^{(2)}m_{H^\pm }^{2} - \delta ^{(2)}m_{W}^{2}, \end{aligned}$$

3.9d $$\begin{aligned} \delta ^{(2)}m_{hH}^{2}&= \frac{1}{2}\left( s_{2\left( \alpha -\beta \right) }\,\delta ^{(2)}m_A^{2} - s_{2\left( \alpha +\beta \right) }\,\delta ^{(2)}m_Z^{2}\right) \nonumber \\&\quad - c_{\beta }^{2}\,\delta ^{(2)}t_{\beta } \left( c_{2(\alpha -\beta )}\,m_A^{2} + c_{2(\alpha +\beta )}\,m_Z^{2}\right) \nonumber \\&\quad + \frac{e}{2\,M_{W}\,s_{\text {w}}} \Biggl [s_{\alpha -\beta }^{3}\,\delta ^{(2)}T_{H} - c_{\alpha -\beta }^{3}\,\delta ^{(2)}T_{h}\Biggr ], \end{aligned}$$

3.9e $$\begin{aligned} \delta ^{(2)}m_{hA}^{2}&= \frac{e}{2\,M_{W}\,s_{\text {w}}}\,s_{\alpha -\beta }\,\delta ^{(2)}T_{A}, \end{aligned}$$

3.9f $$\begin{aligned} \delta ^{(2)}m_{HA}^{2}&= -\frac{e}{2\,M_{W}\,s_{\text {w}}}\,c_{\alpha -\beta }\,\delta ^{(2)}T_{A}. \end{aligned}$$

The entries of $\delta ^{(2)}\mathbf {M}_{hHA}$ that are not listed here are determined by symmetry. When replacing $\delta ^{(2)} \rightarrow \delta$ they are formally equal to the one-loop counterterms listed in Eqs. (53) of Ref. [10] (up to the different convention for the counterterm of $t_\beta$ used there).

**Fig. 2:**
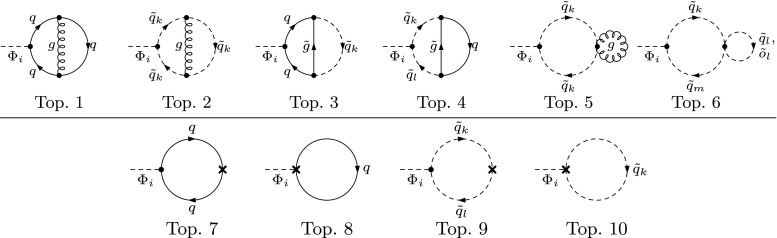
Types of two-loop tadpole diagrams contributing to $T^{(2)}_i$. One-loop counterterm insertions are denoted by a cross. $\Phi _{i} = h,\,H,\,A$; $\tilde{q} \ne \tilde{o}$. Topology 5 contains a one-point loop with a mass-less gluon and is therefore equal to zero

**Fig. 3:**
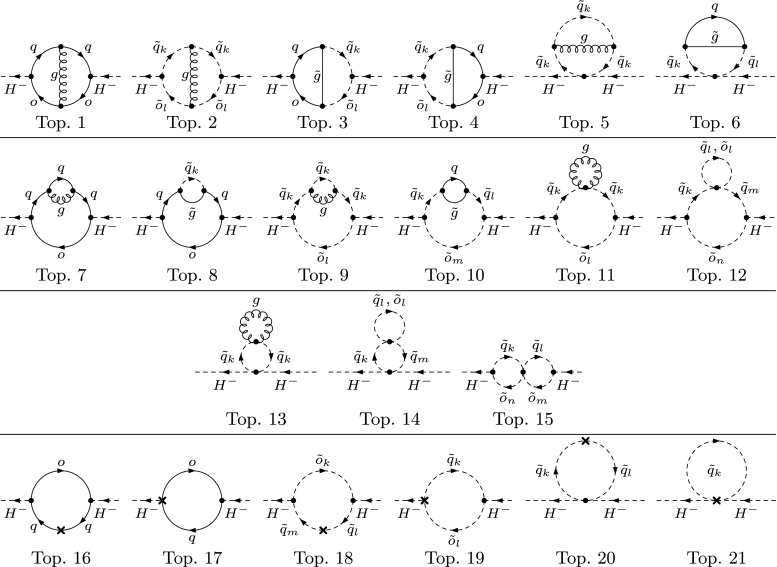
Types of two-loop self-energy diagrams for the charged Higgs bosons. One-loop counterterm insertions are denoted by a cross. $\;q \ne o$, $\;\tilde{q} \ne \tilde{o}$. Topologies 11 and 13 contain a one-point loop with a mass-less gluon and are therefore equal to zero

**Fig. 4:**
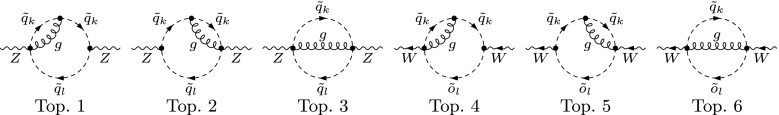
Additional types of two-loop self-energy diagrams for the gauge bosons besides the ones in analogy to Figs. 1 and 3. $\;\tilde{q} \ne \tilde{o}$

The two-loop renormalization constants of Eqs. (3.2)–(3.9) are fixed by extending the renormalization scheme of Ref. [10] from the one-loop to the two-loop order:

- The tadpole counterterms $\delta ^{(2)} T_i$ are fixed by requiring that the minimum of the Higgs potential is not shifted, which means that the tadpole coefficients have to vanish at each order. At the two-loop level, the condition reads
  3.10 $$\begin{aligned} T_{i}^{(2)} + \delta ^{(2)}T_{i} = 0, \quad i = h,\,H,\,A, \end{aligned}$$
  where the $T_{i}^{(2)}$ denote the unrenormalized one-point functions at two-loop order, see Fig. 2 for the contributing two-loop diagrams. The aforementioned relation for the mixing angles $\beta _{n} = \beta _{c} = \beta$ is a consequence of the tadpole conditions $T_i = 0$ at lowest order.
- The charged Higgs-boson mass $m_{H^\pm }$ is the only independent mass parameter of the Higgs sector and is used as an input quantity. Accordingly, the corresponding mass counterterm is fixed by an independent renormalization condition, chosen as on-shell, given by
  3.11 $$\begin{aligned} \mathfrak {R}\mathfrak {e}{\left[ \hat{\Sigma }_{H^{\pm }}^{(2)}(m_{H^{\pm }}^2)\right] } = 0. \end{aligned}$$
  The renormalized charged-Higgs self-energy at the two-loop level can be expressed in terms of the unrenormalized charged self-energy and its respective counterterms
  3.12 $$\begin{aligned} \hat{\Sigma }_{H^\pm }^{(2)} (m_{H^{\pm }}) = \Sigma _{H^\pm }^{(2)} (m_{H^{\pm }}^2) - \delta ^{(2)}m^2_{H^{\pm }}, \end{aligned}$$
  leading to the mass counterterm
  3.13 $$\begin{aligned} \delta ^{(2)}m_{H^{\pm }}^{2} = \mathfrak {R}\mathfrak {e}{\left[ \Sigma _{H^\pm }^{(2)}{\left( m_{H^{\pm }}^2\right) }\right] } \end{aligned}$$
  when applying the on-shell condition. The contributing Feynman diagrams are shown in Fig. 3. As we neglect flavor mixing *q*, *o*, $\tilde{q}$ and $\tilde{o}$ always belong to the same generation. As a consequence, the vertices with four squarks in topologies 12, 14 and 15 are only non-zero when all adjacent fields are of the same generation.
- The field-renormalization constants of the Higgs mass eigenstates in Eq. (3.6) are combinations of the basic doublet-field renormalization constants $\delta ^{(2)}Z_{\mathcal {H}_{1}}$ and $\delta ^{(2)}Z_{\mathcal {H}_{2}}$, which are fixed by the UV-divergent parts of the derivatives of the corresponding self-energies,
  3.14 $$\begin{aligned} \delta ^{(2)}Z_{\mathcal {H}_{1}}= & {} -\left[ \frac{d\Sigma ^{(2)}_{HH}(p^2)}{dp^2} \right] _{\alpha =0}^{\text {div}} ,\nonumber \\ \delta ^{(2)}Z_{\mathcal {H}_{2}}= & {} -\left[ \frac{d\Sigma ^{(2)}_{hh}(p^2)}{dp^2} \right] _{\alpha =0}^{\text {div}} . \end{aligned}$$
- Also $t_\beta$ is renormalized by a purely UV-divergent counterterm, which was shown to be a convenient choice [105] (see also Refs. [106, 107]). Alternative process-dependent definitions for the renormalization of $t_{\beta }$ can be found in Ref. [108]. For the class of two-loop corrections of $\mathcal {O}{\left( \alpha \alpha _s\right) }$ the counterterm can be written as
  3.15 $$\begin{aligned} \delta ^{(2)}t_{\beta }^2&= t_{\beta }^2 \left( \delta ^{(2)}Z_{\mathcal {H}_{2}} - \delta ^{(2)}Z_{\mathcal {H}_{1}}\right) . \end{aligned}$$
- When neglecting momentum-dependent contributions and taking the gaugeless limit, the purely UV-divergent two-loop counterterms $\delta ^{(2)}Z_{\mathcal {H}_{1}}$, $\delta ^{(2)}Z_{\mathcal {H}_{2}}$ and $\delta ^{(2)}t_{\beta }$ cancel each other and are therefore not required for renormalization, compare Ref. [75]. If one of these two limitations is dropped, $\delta ^{(2)}Z_{\mathcal {H}_{1}}$, $\delta ^{(2)}Z_{\mathcal {H}_{2}}$ and $\delta ^{(2)}t_{\beta }$ are necessary in order to obtain a UV-finite result. In the corrections discussed in this article these counterterms have to be taken into account as none of these approximations is used.
  It should also be noted that the chosen renormalization conditions for $\delta ^{(2)}Z_{\mathcal {H}_{2}}$ and $\delta ^{(2)}t_{\beta }$ are not equal to pure $\overline{\text {DR}}$ conditions, since the top-mass counterterm $\delta ^{(1)}m_t$ which enters in $\delta ^{(2)}Z_{\mathcal {H}_{2}}$ is fixed by an on-shell condition. The resulting differences between the two schemes have been discussed in [54, 55].
- Renormalization of the *D* terms in the Higgs–squark couplings which are induced by the gauge coupling $g_2$, as well as the relation between the charged and *CP*-odd Higgs masses require counterterms for the *Z*- and *W*-boson masses, $\delta ^{(2)}M_Z^2$ and $\delta ^{(2)}M_W^2$, respectively. We treat $M_W$ and $M_Z$ as independent input parameters and fix their renormalization constants by the on-shell conditions
  3.16 $$\begin{aligned} \mathfrak {R}\mathfrak {e}{\left[ \hat{\Sigma }_{Z,W}^{(2)}(M_{Z,W}^2)\right] }&= 0 \,, \end{aligned}$$
  leading to
  3.17 $$\begin{aligned} \delta ^{(2)}M_{Z,W}^{2} = \mathfrak {R}\mathfrak {e}{\left[ \Sigma _{Z,W}^{(2)}{\left( M_{Z,W}^2\right) }\right] }. \end{aligned}$$
  Here $\Sigma _{Z,W}^{(2)}$ denote the transverse parts of the two-loop self-energies of *Z* and *W*, respectively.
  Most of the Feynman diagrams contributing to the two-loop self-energies $\Sigma _{Z,W}^{(2)}$ differ from the Higgs self-energies depicted in Figs. 1 and 3 only by the external fields. Pictorially, they can be obtained by replacing the neutral external Higgs fields by the *Z*, and the charged Higgs field by the *W* boson field. All additional topologies are depicted in Fig. 4.

### Sub-loop renormalization

Apart from the genuine two-loop diagrams, the lowest-order QCD contributions to the self-energies and tadpoles involve one-loop diagrams with insertions of one-loop counterterms. This subrenormalization concerns masses and mixing of the colored particles.

The required one-loop counterterms for subrenormalization arise from the quark *q* and scalar quark $\tilde{q}$ sectors. The squark mass matrices in the $\big (\tilde{q}_{\text {L}},\,\tilde{q}_{\text {R}}\big )$ bases are given in lowest order by

3.18 $$\begin{aligned} \mathbf {M}_{\tilde{q}}&= \begin{pmatrix} m_{\tilde{q}_{\text {L}}}^{2} + m_{q}^{2} + M_Z^2\,c_{2\beta } (T_q^3 - Q_q\,s^2_{\mathrm {w}}) &{} m_{q}\left( A_{q}^{*} - \mu \,\kappa _q \right) \\ m_{q}\left( A_{q} - \mu ^{*}\,\kappa _q \right) &{} m_{\tilde{q}_{\text {R}}}^{2} + m_{q}^{2} + M_Z^2\,c_{2\beta }\,Q_q\,s^2_{\mathrm {w}} \end{pmatrix},&\begin{aligned} \kappa _{t,c,u}&= \frac{1}{t_{\beta }},\\ \kappa _{b,s,d}&= t_{\beta }, \end{aligned} \end{aligned}$$

with $Q_q$ and $T^3_q$ denoting charge and isospin of $q \in \{u, c, t, d, s, b\}$. For the sake of convenience we suppress repeating the indices of the first and second generation in the following since renormalization is analogous to the third generation. *SU*(2)-invariance requires $m_{\tilde{t}_{\text {L}}}^{2} = m_{\tilde{b}_{\text {L}}}^{2} \equiv m_{\tilde{Q}_{3}}^{2}$.

The squark mass eigenvalues can be obtained from unitary transformations,

3.19 $$\begin{aligned} \mathbf {U}^{\tilde{q}}\mathbf {M}_{\tilde{q}}\mathbf {U}^{\tilde{q}\dagger } = {\mathrm {diag}}{\left( m_{\tilde{q}_{1}}^{2}, m_{\tilde{q}_{2}}^{2}\right) }. \end{aligned}$$

Since $A_{q}$ and $\mu$ are complex parameters, the unitary matrices $\mathbf {U}^{\tilde{q}}$ can be described by the mixing angle $\theta _{\tilde{q}}$ and an additional phase $\varphi _{\tilde{q}}$.

The independent parameters which enter the two-loop calculation through the quark–squark sector are: the quark masses $m_q$, the soft SUSY-breaking parameters $m_{\tilde{Q}_{i}}$ and $m_{\tilde{q}_{\text {R}}}$, and the complex trilinear couplings $A_{q} = |A_{q}|\,\mathrm {e}^{i\phi _{A_{q}}}$. These parameters have to be renormalized at the one-loop level,

3.20 $$\begin{aligned} m_q&\rightarrow m_q + \delta ^{(1)}m_q,\quad m_{\tilde{q}_\text {L,R}}^2 \rightarrow m_{\tilde{q}_\text {L,R}}^2 + \delta ^{(1)}m_{\tilde{q}_\text {L,R}}^2,\nonumber \\ A_q&\rightarrow A_q + \delta ^{(1)}A_q, \end{aligned}$$

thus defining transformations $\mathbf {M}_{\tilde{q}}\rightarrow \mathbf {M}_{\tilde{q}} + \delta ^{(1)}\mathbf {M}_{\tilde{q}}$ for the mass matrices in Eq. (3.18). The other free parameter $\mu$, which is related to the Higgsino sector, enters the self-energies as well. However, the renormalization of $\mu$ does not receive one-loop corrections of $\mathcal {O}{\left( \alpha _s\right) }$ and is therefore not part of the contributions considered in this calculation.

**Fig. 5:**
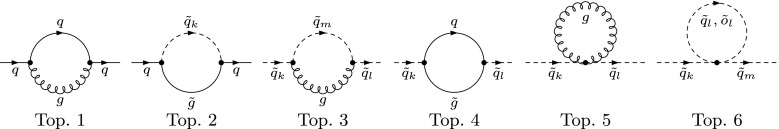
Types of Feynman diagrams for the renormalization of the quark–squark sector. $\;\tilde{q} \ne \tilde{o}$. Topology 5 contains a one-point loop with a mass-less gluon and is therefore equal to zero

The individual renormalization conditions for the colored sector are formulated as follows:

- Renormalization of the top quark mass is carried out in the on-shell scheme, i.e.
  3.21 $$\begin{aligned} \delta ^{(1)}m_{t}&= m_{t} \,\nonumber \\&\quad \mathfrak {R}\mathfrak {e}{\left[ \frac{1}{2} \left( \Sigma _{t}^{\text {L}(1)}{\left( m_{t}^{2}\right) } + \Sigma _{t}^{\text {R}(1)}{\left( m_{t}^{2}\right) }\right) + \Sigma _{t}^{\text {S}(1)}{\left( m_{t}^{2}\right) }\right] }, \end{aligned}$$
  where the quark self-energy is given in terms of its Lorentz decomposition 3.22 with the left-, right-handed projectors $\omega _{-,+}=\tfrac{1}{2}\left( 1\mp \gamma _5\right)$.
  The bottom mass is renormalized in the $\overline{\text {DR}}$ scheme (see Refs. [41, 42, 109]) at the scale $m_t^{\text {os}}$. The counterterm can be obtained by using the expression in analogy to the counterterm for the top quark mass in Eq. (3.21) and restricting to the UV-divergent contributions at the scale $m_t^{\text {os}}$. The choice of a $\overline{\text {DR}}$ renormalization for $m_b$ is convenient in order to incorporate a resummation of $\tan \beta$-enhanced contributions to the relation between the bottom quark mass and the bottom Yukawa coupling, see Sect. 3.3 below. The contributing Feynman diagrams for the renormalization of $m_t$ and $m_b$ are depicted in Fig. 5.
- In order to fix the renormalization constants of the stop sector, we employ the relation
  3.23 $$\begin{aligned} \delta ^{(1)}\mathbf {M}_{\tilde{t}}&= \delta ^{(1)}{\left( \mathbf {U}^{\tilde{t}\dagger }\,{\mathrm {diag}}{\left( m_{\tilde{q}_{1}}^{2},\, m_{\tilde{q}_{2}}^{2}\right) }\,\mathbf {U}^{\tilde{t}}\right) } \nonumber \\ {}&= \mathbf {U}^{\tilde{t}\dagger }\begin{pmatrix} \delta ^{(1)}m_{\tilde{t}_{1}}^{2} &{} \delta ^{(1)}m_{\tilde{t}_{1}\tilde{t}_{2}}^{2} \\ \delta ^{(1)}m_{\tilde{t}_{1}\tilde{t}_{2}}^{2\,*} &{} \delta ^{(1)}m_{\tilde{t}_{2}}^{2} \end{pmatrix}\mathbf {U}^{\tilde{t}}. \end{aligned}$$
  Thus we derive
  3.24a $$\begin{aligned} \delta ^{(1)}m_{\tilde{t}_{\text {L}}}^2&= \sum \limits _{i=1}^2|\mathbf {U}^{\tilde{t}}_{i1}|^{2}\,\delta ^{(1)}m_{\tilde{t}_{i}}^{2} + 2\,\mathfrak {R}\mathfrak {e}{\left[ \mathbf {U}^{\tilde{t}}_{21}\mathbf {U}^{\tilde{t}*}_{11}\,\delta ^{(1)}m_{\tilde{t}_{1}\tilde{t}_{2}}^{2}\right] } \nonumber \\ {}&\quad - 2\,m_{t}\,\delta ^{(1)}m_{t}, \end{aligned}$$
  3.24b $$\begin{aligned} \delta ^{(1)}m_{\tilde{t}_{\text {R}}}^2&= \sum \limits _{i=1}^2|\mathbf {U}^{\tilde{t}}_{i2}|^{2}\,\delta ^{(1)}m_{\tilde{t}_{i}}^{2} + 2\,\mathfrak {R}\mathfrak {e}{\left[ \mathbf {U}^{\tilde{t}}_{22}\mathbf {U}^{\tilde{t}*}_{12}\,\delta ^{(1)}m_{\tilde{t}_{1}\tilde{t}_{2}}^{2}\right] } \nonumber \\ {}&\quad - 2\,m_{t}\,\delta ^{(1)}m_{t}, \end{aligned}$$
  3.24c $$\begin{aligned} \delta ^{(1)}A_t&= \mathbf {U}^{\tilde{t}}_{11}\mathbf {U}^{\tilde{t}*}_{12}\frac{\delta ^{(1)}m_{\tilde{t}_{1}}^{2} - \delta ^{(1)}m_{\tilde{t}_{2}}^{2}}{m_t} + \mathbf {U}^{\tilde{t}}_{21}\mathbf {U}^{\tilde{t}*}_{12}\frac{\delta ^{(1)}m_{\tilde{t}_{1}\tilde{t}_{2}}^{2}}{m_t} \nonumber \\&\quad + \mathbf {U}^{\tilde{t}}_{22}\mathbf {U}^{\tilde{t}*}_{11}\frac{\delta ^{(1)}m_{\tilde{t}_{1}\tilde{t}_{2}}^{2\,*}}{m_t} - \left( A_{t} - \frac{\mu ^{*}}{t_\beta }\right) \frac{\delta ^{(1)}m_t}{m_t}\,. \end{aligned}$$
  The counterterm $\delta ^{(1)}A_t$ given in Eq. (3.24c) provides the renormalization of the complex parameter $A_t$. It should be noted that the counterterm in fact only contributes to the absolute value of $A_t$, while the phase of $A_t$ remains unrenormalized.
  The counterterms $\delta ^{(1)}m_{\tilde{t}_{1}}^{2}$ and $\delta ^{(1)}m_{\tilde{t}_{2}}^{2}$ are fixed by on-shell conditions for the top-squarks,
  3.25 $$\begin{aligned} \delta ^{(1)}m_{\tilde{t}_{i}}^{2}&= \mathfrak {R}\mathfrak {e}{\left[ \, \Sigma _{\tilde{t}_{ii}}^{(1)}{\left( m_{\tilde{t}_{i}}^{2}\right) }\right] }, \quad i=1,2, \end{aligned}$$
  involving the diagonal $\tilde{t}_{1,2}$ self-energies, see Fig. 5. The remaining counterterm $\delta ^{(1)}m_{\tilde{t}_{1}\tilde{t}_{2}}^{2}$ is fixed by the renormalization condition (see Ref. [72])
  3.26 $$\begin{aligned} \delta ^{(1)}m_{\tilde{t}_{1}\tilde{t}_{2}}^{2}&= \frac{1}{2}\,\mathfrak {R}\mathfrak {e}{\left[ \Sigma _{\tilde{t}_{12}}^{(1)}{\left( m_{\tilde{t}_{1}}^{2}\right) } + \Sigma _{\tilde{t}_{12}}^{(1)}{\left( m_{\tilde{t}_{2}}^{2}\right) }\right] }, \end{aligned}$$
  which involves the non-diagonal squark self-energy shown in Fig. 5 with incoming $\tilde{t}_2$ and outgoing $\tilde{t}_1$.
- Between the gauge and mass eigenstates of the bottom squarks we employ an analogous relation to Eq. (3.23). We derive 3.27a
3.27b
3.27c As indicated by the subscript, we choose to renormalize $A_b$ in the $\overline{\text {DR}}$ scheme, which has been shown to be convenient for reasons of numerical stability [41, 42, 109]. The scale of $A_b$ is chosen to be $m_t^{\text {os}}$.
  As a consequence of *SU*(2) invariance the counterterm $\delta ^{(1)}m_{\tilde{b}_{\text {L}}}^{2}$ is not independent, but a derived quantity which is fixed by the renormalization of the top–stop sector in Eq. (3.24a), since
  3.28 $$\begin{aligned} \delta ^{(1)}m_{\tilde{b}_{\text {L}}}^{2} = \delta ^{(1)}m_{\tilde{Q}_{3}}^{2} = \delta ^{(1)}m_{\tilde{t}_{\text {L}}}^{2}\,. \end{aligned}$$
  Inserting Eq. (3.27a) and solving for $\delta ^{(1)}m_{\tilde{b}_1}^{2}$ yields
  3.29 $$\begin{aligned} \delta ^{(1)}m_{\tilde{b}_{1}}^{2}&= \frac{1}{|\mathbf {U}^{\tilde{b}}_{11}|^{2}}\left( \delta ^{(1)}m_{\tilde{t}_{\text {L}}}^{2} - |\mathbf {U}^{\tilde{b}}_{12}|^{2}\,\delta ^{(1)}m_{\tilde{b}_{2}}^{2}\right. \nonumber \\&\quad \left. - 2\,\mathfrak {R}\mathfrak {e}{\left[ \mathbf {U}^{\tilde{b}}_{21}\mathbf {U}^{\tilde{b}*}_{11}\,\delta ^{(1)}m_{\tilde{b}_{1}\tilde{b}_{2}}^{2}\right] } - 2\,m_{b}\,\delta ^{(1)}m_{b}\right) . \end{aligned}$$
  The other two counterterms $\delta ^{(1)}m_{\tilde{b}_{2}}^{2}$ and $\delta ^{(1)}m_{\tilde{b}_{1}\tilde{b}_{2}}^{2}$ are fixed analogously as for the stops:
  3.30a $$\begin{aligned} \delta ^{(1)}m_{\tilde{b}_2}^{2}&= \mathfrak {R}\mathfrak {e}{\left[ \, \Sigma _{\tilde{b}_{22}}^{(1)}{\left( m_{\tilde{b}_2}^{2}\right) }\right] }, \end{aligned}$$
  3.30b $$\begin{aligned} \delta ^{(1)}m_{\tilde{b}_{1}\tilde{b}_{2}}^{2}&= \frac{1}{2}\,\mathfrak {R}\mathfrak {e}{\left[ \Sigma _{\tilde{b}_{12}}^{(1)}{\left( m_{\tilde{b}_{1}}^{2}\right) } + \Sigma _{\tilde{b}_{12}}^{(1)}{\left( m_{\tilde{b}_{2}}^{2}\right) }\right] }. \end{aligned}$$
  Therefore in our scheme only $m_{\tilde{b}_2}$ is renormalized on-shell, while the counterterm $\delta ^{(1)}m_{\tilde{b}_1}^{2}$ is a derived quantity according to Eq. (3.29).

### Resummation of $\tan \beta$-enhanced terms

The Yukawa coupling of the bottom quark $h_b$ receives radiative corrections proportional to $\tan \beta$. Those $\tan \beta$-enhanced contributions can be resummed as described in Refs. [80, 110–115]. The resummed contributions $\Delta _b$ are UV finite and generally yield complex numerical results. For the numerical evaluation in Sect. 5, we use the version for $\Delta _b$ at the one-loop order which is implemented in FeynHiggs and outlined in the following. The largest $\tan \beta$-enhanced contributions can be absorbed by using an effective bottom-quark mass, which is related to the $\overline{\text {DR}}$-renormalized bottom quark mass in the MSSM as follows,

3.31 $$\begin{aligned} m_b^{\overline{\text {DR}},\text {MSSM}}{\left( m_t^{\text {os}}\right) } \simeq m_{b,\text {eff}}&= \frac{m_b^{\overline{\text {DR}},\text {SM}}{\left( m_t^{\text {os}}\right) }}{|1 + \Delta _b |}\left( 1 - \delta _b\right) , \end{aligned}$$

where $m_b^{\overline{\text {DR}},\text {SM}}(m_t^{\text {os}})$ is the bottom mass in the $\overline{\text {DR}}$ renormalization scheme in the Standard Model evaluated at the on-shell top mass. The $\tan \beta$-enhanced contributions are captured in $\Delta _b$ and properly resummed by including them in the denominator. The remaining parts of the scalar part of the $\overline{\text {DR}}$-renormalized bottom self-energy $\hat{\Sigma }_b^{\text {S}}$ which are not enhanced by $\tan \beta$ are contained in $\delta _b$ such that

3.32 $$\begin{aligned} \hat{\Sigma }_b^{\text {S}}{\left( 0\right) } = -\Delta _b - \delta _b. \end{aligned}$$

The expression $\Delta _b$ at the one-loop order contains contributions from gluinos, charginos and neutralinos (ordered in decreasing numerical importance) and reads

3.33 $$\begin{aligned} \begin{aligned} \Delta _b&= \frac{2\,\alpha _s{\left( Q\right) }}{3\pi }\frac{M_3^*}{m_b}\sum \limits _{i=1}^2\mathbf {U}^{\tilde{b}}_{i1}\mathbf {U}^{\tilde{b}*}_{i2}\,B_{0}{\left( 0,|M_3|^2,m_{\tilde{b}_i}^2\right) }\\&\quad +\frac{\alpha {\left( Q\right) }}{4\pi }\sum \limits _{g=1}^3\sum \limits _{i,j=1}^2\frac{m_{\tilde{\chi }^\pm _i}}{m_b} c_{\text {L}} c_{\text {R}} |\mathbf {C}_{g3}|^2\,B_{0}{\left( 0,m_{\tilde{\chi }^\pm _i}^2,m_{\tilde{u}^g_j}^2\right) }\\&\quad -\frac{\alpha {\left( Q\right) }}{8\pi }\sum \limits _{i=1}^4\sum \limits _{j=1}^2 \frac{m_{\tilde{\chi }^0_i}}{m_b} n_{\text {L}} n_{\text {R}} B_{0}{\left( 0,m_{\tilde{\chi }^0_i}^2,m_{\tilde{b}_j}^2\right) }\ . \end{aligned} \end{aligned}$$

The couplings $\alpha _s$ and $\alpha$ are running parameters and are evaluated at the scale $Q = \sqrt{\phantom {Q}m_{\tilde{b}_1}m_{\tilde{b}_2}}$. The symbol $\mathbf {C}$ depicts the CKM matrix, and $u^g,\,\tilde{u}^g$ are the $g\hbox {th}$ generation up-type quarks and squarks, whereas $B_{0}{\left( 0, m_1, m_2\right) }$ and $B_{1}{\left( 0, m_1, m_2\right) }$ are one-loop functions.^5^ As mentioned above, we otherwise neglect CKM mixing in the two-loop contributions that we evaluate. The renormalization scale $\mu _{r}$ from the loop integrals drops out. The coefficients $c_{\text {L,R}}$ and $n_{\text {L,R}}$ are given by

3.34a $$\begin{aligned} c_{\text {L}}&= \frac{\mathbf {V}^{\tilde{\chi }*}_{i1}\,\mathbf {U}^{\tilde{u}^g}_{j1}}{s_{\text {w}}} - \frac{m_{u^g}\,\mathbf {V}^{\tilde{\chi }*}_{i2}\,\mathbf {U}^{\tilde{u}^g}_{j2}}{\sqrt{2}\,M_W\,s_\beta \,s_{\text {w}}},\nonumber \\ c_{\text {R}}&= \frac{m_b\,\mathbf {U}^{\tilde{\chi }*}_{i2}\,\mathbf {U}^{\tilde{u}^g*}_{j1}}{\sqrt{2}\,M_W\,c_\beta \,s_{\text {w}}}\,, \end{aligned}$$

3.34b $$\begin{aligned} n_{\text {L}}&= \left( \frac{\mathbf {N}^{\tilde{\chi }*}_{i1}}{3\,c_{\text {w}}} - \frac{\mathbf {N}^{\tilde{\chi }*}_{i2}}{s_{\text {w}}}\right) \mathbf {U}^{\tilde{b}}_{j1} + \frac{m_b\,\mathbf {N}^{\tilde{\chi }*}_{i3}\,\mathbf {U}^{\tilde{b}}_{j2}}{M_W\,c_\beta \,s_{\text {w}}},\nonumber \\ n_{\text {R}}&= \frac{2\,\mathbf {N}^{\tilde{\chi }*}_{i1}\,\mathbf {U}^{\tilde{b}*}_{j2}}{3\,c_{\text {w}}} + \frac{m_b\,\mathbf {N}^{\tilde{\chi }*}_{i3}\,\mathbf {U}^{\tilde{b}*}_{j1}}{M_W\,c_\beta \,s_{\text {w}}}\,. \end{aligned}$$

In order to obtain a full conversion of the bottom mass between the on-shell scheme and the $\overline{\text {DR}}$ scheme in Eq. (3.31), those parts of the bottom self-energy which are not enhanced by $\tan \beta$ are included in $\delta _b$ and incorporated in the numerator of Eq. (3.31).

At the one-loop order they read

3.35 $$\begin{aligned} \begin{aligned} \delta b&= \frac{\alpha _s{\left( Q\right) }}{3\pi }\sum \limits _{i=1}^2B_{1}{\left( 0,|M_3|^2,m_{\tilde{b}_i}^2\right) } \\&\quad + \frac{\alpha {\left( Q\right) }}{8\pi }\sum \limits _{g=1}^3\sum \limits _{i,j=1}^2\left[ \left| c_{\text {L}}\right| ^2 + \left| c_{\text {R}}\right| ^2\right] |\mathbf {C}_{g3}|^2\,\\&\quad B_{1}{\left( 0,m_{\tilde{\chi }^\pm _i}^2,m_{\tilde{u}^g_j}^2\right) }\\&\quad + \frac{\alpha {\left( Q\right) }}{16\pi }\sum \limits _{i=1}^4\sum \limits _{j=1}^2\left[ \left| n_{\text {L}}\right| ^2 + \left| n_{\text {R}}\right| ^2\right] B_{1}{\left( 0,m_{\tilde{\chi }^0_i}^2,m_{\tilde{b}_j}^2\right) }\ . \end{aligned} \end{aligned}$$

The parameters entering in $\Delta _b$ and $\delta _b$ are computed in the limit of large $\tan \beta$. The chargino and neutralino masses and mixing matrices are then obtained as

3.36 $$\begin{aligned} \lim _{t_\beta \rightarrow \infty } \text {diag}{\left( m_{\tilde{\chi }^\pm _1},m_{\tilde{\chi }^\pm _2}\right) }&= \mathbf {U}^{\tilde{\chi }*}\mathbf {X}\mathbf {V}^{\tilde{\chi }\dagger },\nonumber \\ \lim _{t_\beta \rightarrow \infty } \text {diag}{\left( m_{\tilde{\chi }^0_1},m_{\tilde{\chi }^0_2},m_{\tilde{\chi }^0_3},m_{\tilde{\chi }^0_4}\right) }&= \mathbf {N}^{\tilde{\chi }*}\mathbf {Y}\mathbf {N}^{\tilde{\chi }\dagger }, \end{aligned}$$

where we use

3.37 $$\begin{aligned} \mathbf {X}&= \lim _{t_\beta \rightarrow \infty } \mathbf {M}_{\chi ^\pm } = \begin{pmatrix} M_2 &{} \sqrt{2} M_W s_\beta \\ 0 &{} \mu \end{pmatrix},\nonumber \\ \mathbf {Y}&= \lim _{t_\beta \rightarrow \infty } \mathbf {M}_{\chi ^0} = \begin{pmatrix} M_1 &{} 0 &{} 0 &{} M_Z s_\beta s_{\text {w}}\\ 0 &{} M_2 &{} 0 &{} -M_Z s_\beta c_{\text {w}}\\ 0 &{} 0 &{} 0 &{} -\mu \\ M_Z s_\beta s_{\text {w}} &{} -M_Z s_\beta c_{\text {w}} &{} -\mu &{} 0\end{pmatrix}. \end{aligned}$$

Thereby the matrices $\mathbf {U}^{\tilde{\chi }}$ and $\mathbf {V}^{\tilde{\chi }}$ yield a singular value decomposition for $\mathbf {X}$, and the matrix $\mathbf {N}^{\tilde{\chi }}$ yields Takagi’s factorization [116] on $\mathbf {Y}$.

The sbottom masses in this limit are computed from the matrix given in Eq. (3.18) at $A_b = 0$. Since the bottom mass itself also enters that matrix, the final solution for $m_{b,\text {eff}}$ is found iteratively.

By using Eq. (3.31) for the bottom mass in the one-loop contributions to the Higgs masses, the leading higher-order corrections to the Higgs masses from the bottom–sbottom sector are generated. The contributions of the bottom–sbottom sector to the two-loop self-energies presented in this article add further subleading shifts. It should be noted that the expression given in Eq. (3.31), which employs the $\overline{\text {DR}}$ scheme in the MSSM, is chosen such that no double counting of the terms contained in $m_{b,\text {eff}}$ occurs at the two-loop level.

**Fig. 6:**
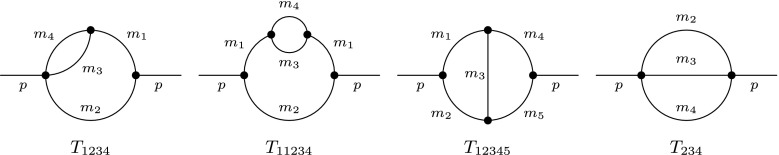
Irreducible two-loop topologies resulting from tensor reduction, calculated numerically with the program SecDec. Some of the internal lines may also be massless

## Numerical evaluation of the self-energies

The renormalized two-loop self-energies are expressed in terms of two-loop two-point multi-scale integrals with non-zero external momenta. With the help of TwoCalc [92] and Reduze [103] all integrals can be reduced to the four irreducible scalar two-loop topologies depicted in Fig. 6, and products of analytically well-known one-loop one- and two-point functions.

The scalar two-loop integrals are defined as

4.1 $$\begin{aligned}&T_{i_1 i_2\cdots i_n}(p^2, m_{i_1}^2,m_{i_2}^2,\ldots , m_{i_5}^2) = \left( 2 \pi \mu _r \right) ^{2(4-D)} \times \iint \frac{\text {d}^D q_1}{i \pi ^2} \nonumber \\&\quad \, \frac{\text {d}^D q_2}{i \pi ^2} \frac{1}{(k_{i_1}^2-m_{i_1}^2+ i \delta )(k_{i_2}^2-m_{i_2}^2+ i \delta )\cdots (k_{i_n}^2-m_{i_n}^2+ {i}\delta )} \text { ,} \nonumber \\ \end{aligned}$$

where *p* is the external momentum, $q_i$ are the loop momenta, $m_i$ the masses of the propagators, $\mu _r$ is the renormalization scale and $D=4-2\varepsilon$ the dimension. The ${i}\delta$ results from the solutions of the field equations in terms of causal Green functions, while the indices ${i_1, i_2, \ldots i_n}$ label which $k_{i}$ and $m_i$ appear in the propagators of the integral. The five different $k_{i}$ read

4.2 $$\begin{aligned} k_1&=q_1, \quad k_2=q_1+p, \quad k_3=q_2 -q_1,\nonumber \\ k_4&=q_2, \quad k_5=q_2+p. \end{aligned}$$

The irreducible two-loop integrals of Fig. 6 may depend on up to five different internal mass scales taken from the following set,

4.3 $$\begin{aligned} m_t,\, m_b,\, m_{\tilde{t}_1},\, m_{\tilde{t}_2},\, m_{\tilde{b}_1},\, m_{\tilde{b}_2},\, m_{\tilde{g}} = |M_3|, \end{aligned}$$

in addition to a non-zero external momentum, taking the values of $p^2=M_{h_1}^2,\, M_{h_2}^2,\, M_{h_3}^2$ when entering the unrenormalized self-energies, or $p^2=m_{H^\pm }^2,\, m_W^2,\, m_Z^2$ when entering the self-energies through two-loop renormalization constants. Recently, a lot of progress has been made towards describing and evaluating integrals of this class analytically [117–126]. However, to the best of our knowledge, an implementation of the analytical results for all topologies in Fig. 6 is not publicly available. We have therefore calculated these integrals numerically using the program SecDec [94–96].

For the evaluation, the resulting new contributions to the neutral Higgs-boson self-energies have been added to FeynHiggs via its interface to external programs, see section 2.4 of Ref. [53] for details. We have extended the existing interface to the program SecDec in FeynHiggs to deal with the 177 mass configurations of which 88 are computed at four different kinematic points, 72 at two and the rest at one kinematic point. The parameters entering the integrals are evaluated by FeynHiggs and passed on to SecDec. It should be noted that the heavy growth of mass configurations with respect to non-electroweak corrections is due to an increase in the number of mass scales involved in the renormalized self-energies.

We constructed two independent integration setups to allow for consistency checks of the numerical result. The two-point one-loop topologies entering the self-energies up to $\mathcal {O}(\varepsilon )$ are known analytically. The bulk of their implementation was previously tested in Ref. [53] and compared with the authors of Ref. [54]. Additional mass configurations were newly implemented and checked against SecDec. The increase in two-loop mass configurations by more than a factor five with respect to the previous setup in Ref. [53] calls for a higher precision of the integrals to avoid numerical instabilities due to cancellations. With the integral reduction, unphysical thresholds can be introduced which cancel in the sum of all contributing diagrams. Numerically, due to round-off errors, the cancellation might however not always be exact, leading to numerical instabilities. The latter are cured by introducing a small imaginary part to the denominators of the coefficients arising from the integral reduction. We have verified that the numerical dependence of the self-energies on this technical regularization parameter is negligible.

The fact that we take a non-zero value of the bottom quark mass into account leads to a large hierarchy among the different mass scales. Numerical convergence at the desired accuracy is therefore difficult to accomplish. On the other hand, we have analyzed the influence of the quark masses of the first and second generation on the two-loop integrals in the self-energies. For the second generation and $\tan {\beta }\gg 1$ a negative shift in the Higgs-boson mass correction of only about 20 MeV can be observed when neglecting the light quark masses. The effect is even smaller for the quark masses of the first generation. The terms which involve the light quark masses in couplings are negligible, too. It is due to this reason that we will assume the first and second generation quarks to be massless throughout the rest of our numerical analysis. The numerical impact of the gauge contributions of the light quarks will be discussed below.

In order to achieve a relative precision of at least $10^{-7}$ for each integral, we use the deterministic integrator Cuhre included in the Cuba library [127, 128] but have optimized the integration parameters for each integral topology and mass configuration individually.

As a further crosscheck of our computation, we have compared the $\mathcal {O}{\left( \alpha \alpha _s\right) }$ contribution by the top–stop and bottom–sbottom particles to the *Z*-boson self-energy which is required for renormalization of the Higgs sector. Since [54] uses massless bottom quarks, we have reevaluated our result for the *Z* self-energy in the limit $m_b=0$. In order to avoid a dependence on the renormalization scheme of the quark–squark sectors, the *Z*-boson self-energy has been evaluated in the $\overline{\text {DR}}$ scheme by both groups for this comparison. Overall we have found a very good agreement with discrepancies at the level of 0.3 GeV$^2$.

We find an overall uncertainty of the self-energies entering the light Higgs-boson mass of maximally $0.2\%$ by adding all uncertainties on the numerical evaluation of the two-loop integrals in quadrature. Given the resulting size of our newly computed corrections analyzed in the next section, the absolute uncertainty on the light Higgs boson mass is maximally 0.4 MeV, which is well below the shift coming from neglecting light quark masses from the first and second generation.

The total of 513 integrals have been computed numerically on the fly before passing the resulting two-loop self-energies back to FeynHiggs, where they are added to the corresponding matrix elements just before the determination of the propagator poles.

**Table 1:** Comparison of the results for the light Higgs-boson mass with Refs. [53, 54] for four benchmark scenarios from Refs. [129, 131] with $m_A=500$ GeV and $\tan \beta =20$

Scenario	$m_h^{\text {max}}$	$m_h^{\text {mod}+}$	$m_h^{\text {mod}-}$	Modified light-stop
$M_h^{\text {old}}$ (GeV)	128.31	125.36	124.84	122.68
$M_h^{\text {old}}$ (GeV) [54]	128.32	125.36	124.84	122.67
$M_h^{\text {old}}+\mathcal {O}(\alpha _t\alpha _s p^2)$ (GeV) [53]	128.25	125.23	123.83	122.64
$M_h^{\text {old}}+\mathcal {O}(\alpha _t\alpha _s p^2)$ (GeV) [54]	127.94	124.98	123.96	122.33
$M_h^{\text {old}}+\mathcal {O}(\alpha _t\alpha _s p^2)+\mathcal {O}(\alpha \alpha _s)$ (GeV) [54]	128.38	125.63	124.90	122.46
$M_h^{\text {new}}$ (GeV)	128.53	125.75	124.85	122.61

## Numerical results for the Higgs mass spectrum

In the following we analyze the numerical impact of the newly computed corrections. We start with a comparison with earlier results in the literature and then discuss our results in three different scenarios: an $m_h^{\text {mod}}$-like scenario (based on Ref. [129]), a scenario with a particularly large value of $\tan \beta$ where contributions from the bottom and sbottom sector are enhanced, and a low-$m_H$ scenario (inspired by Refs. [5, 129]). For better readability of the results, we define three different Higgs-boson masses resulting from different higher-order contributions

5.1 $$\begin{aligned}&M_{h_i}^{\text {old}}, \text { contains: } \mathcal {O}(\alpha _t\alpha _s)|_{p^2=0} \text { with complex parameters},\nonumber \\&\tilde{M}_{h_i}^{\text {old}}, \text { contains: } \text {same as } M_{h_i}^{\text {old}} + \left. \mathcal {O}(\alpha _b\alpha _s)\right| _{p^2=0}\nonumber \\&\quad \text { with real parameters},\nonumber \\&M_{h_i}^{\text {new}}, \text { contains: } \mathcal {O}(\alpha _q\alpha _s),\mathcal {O}(\alpha \alpha _s), \mathcal {O}(h_{q}h_{o}\alpha _s)\nonumber \\&\quad \text { with non-zero } p^2,\nonumber \\&\text {with } i \in \{1,2,3\},\quad q,o\in \{b,t\}. \end{aligned}$$

All the above results contain the full one-loop and leading $\mathcal {O}(\alpha _t^2)|_{p^2=0}$ two-loop contributions, and the $\tan \beta$-enhanced contributions to the relation between the bottom quark mass and the bottom Yukawa coupling are resummed, see Sect. 3.3. As mentioned earlier, the quark masses and Yukawa couplings of the first and second family are neglected. Thus, the first and second generation contributes only at $\mathcal {O}(\alpha \alpha _s)$ by D-term contributions of the sfermions. We focus our numerical discussion on the fixed-order result up to the two-loop level, i.e. no combination with resummed higher-order logarithmic contributions as discussed in Refs. [59–61] is employed.

Using the definitions of Eq. (5.1), we assign

5.2 $$\begin{aligned} \Delta M_{h_i} = M_{h_i}^{\text {new}} - M_{h_i}^{\text {old}}, \quad \Delta \tilde{M}_{h_i} = M_{h_i}^{\text {new}} - \tilde{M}_{h_i}^{\text {old}}. \end{aligned}$$

The size of the effects of our newly computed contributions is contained in $\Delta M_{h_i}$, since all the previously known terms are subtracted. So far, the two-loop terms of $\mathcal {O}(\alpha _b\alpha _s)$ were only known in the MSSM with real parameters and $m_A$ as input parameter. $\Delta \tilde{M}_{h_i}$ shows our new contributions without these terms, if $m_A$ is chosen as input parameter.

Below we will discuss our results for non-zero phases of complex parameters. We investigate in particular the variation of the phases $\phi _{M_3},\,\phi _{A_t}$ and $\phi _{A_b}$, which are much less constrained by experimental bounds on EDMs than the phases of $\mu$, $M_1$ (in the usual convention where the parameter $M_2$ is chosen to be real) and the phases of the trilinear couplings of the first and second generation. As discussed e.g. in Ref. [130], scenarios with relatively large phase values are possible. In order to demonstrate the possible impact of the phase variations on the Higgs spectrum, below we display the phase dependences over the whole range $\left( -\pi ,\pi \right]$.

### Comparison with earlier results

In a first step, in Table 1 we show a comparison of the results for the light Higgs-boson mass including our new contributions with the results of Ref. [54], where in the MSSM with real parameters the corrections of $\mathcal {O}(\alpha _t\alpha _s p^2)$ and the full corrections of $\mathcal {O}(\alpha \alpha _s)$ have been evaluated, and with the results up to $\mathcal {O}(\alpha _t\alpha _s p^2)$ in the MSSM with real parameters from Ref. [53]. The comparison is carried out for the benchmark scenarios $m_h^{\text {max}}$, $m_h^{\text {mod}+}$, $m_h^{\text {mod}-}$ defined in Ref. [129] and for a modified light-stop scenario used in Ref. [131]. We find overall good agreement with the results of Ref. [54]. The comparison of the corrections of $\mathcal {O}(\alpha _t\alpha _s p^2)$ with the full corrections of $\mathcal {O}(\alpha \alpha _s)$ shows that the inclusion of momentum dependence in the $\mathcal {O}(\alpha _t\alpha _s p^2)$ corrections yields a downward shift in $M_h$ which is to a large extent compensated by the further corrections of $\mathcal {O}(\alpha \alpha _s)$ for the scenarios that are considered here. The corrections beyond those of $\mathcal {O}(\alpha _t\alpha _s p^2)$ yield an upward shift in $M_h$ of 520 MeV in the $m_h^{\text {mod}+}$ and more than 1 GeV in the $m_h^{\text {mod}-}$ scenario compared to the results of Ref. [53]. The size of the corrections shows a significant dependence on the parameters in the stop sector. The corrections are largest in the $m_h^{\text {mod}-}$ scenario, where the stop masses are near the SUSY scale and $A_t$ is negative. In this case there is a large compensation between the downward shift caused by the corrections of $\mathcal {O}(\alpha _t\alpha _s p^2)$ and the upward shift caused by the further corrections of $\mathcal {O}(\alpha \alpha _s)$. On the other hand, the corrections are smallest for the modified light-stop scenario, in which case we find that the contributions beyond the ones of $\mathcal {O}(\alpha _t\alpha _s p^2)$ from Ref. [53] even yield a small downward shift. The numerical differences between the results for the contributions of $\mathcal {O}(\alpha _t\alpha _s p^2)$ from Refs. [53, 54], which amount up to 0.3 GeV for the examples considered here, result from different renormalization scheme choices of $\delta ^{(2)}Z_{\mathcal {H}_{i}}$, see the discussion in Refs. [53–55]. Those differences in the renormalization schemes also affect the comparison between our results for $M_h^{\text {new}}$ and the results for $M_h^{\text {old}}+\mathcal {O}(\alpha _t\alpha _s p^2)+\mathcal {O}(\alpha \alpha _s)$ from Ref. [54] in Table 1.

**Table 2:** Values for the lightest Higgs-boson mass in the $m_h^{\text {mod}+}$-like and $m_h^{\text {mod}-}$-like scenarios of Ref. [129] using $M_{\text {SUSY}}=2,3$ TeV and $m_A=500$ GeV, $\tan \beta =20$. The results are compared with those provided by the authors of Ref. [54] for two different wave-function renormalization schemes

	This publication	Ref. [54]	
	$\delta ^{(2)}Z_{\mathcal {H}_{i}}^{{\mathrm{Ref. }}\,[53]}$	$\delta ^{(2)}Z_{\mathcal {H}_{i}}^ {{\mathrm{Ref. }}\,[53]}$	$\delta ^{(2)}Z_{\mathcal {H}_{i}}^{{\mathrm{Ref. }}\, [54]}$
$m_h^{\text {mod}+}$-like	$M_{\text {SUSY}}=2$ TeV		
$M_h^{\text {old}}$ (GeV)	129.38	129.38	129.38
$M_h^{\text {new}}$ (GeV)	129.92	129.92	129.84
	$M_{\text {SUSY}}=3$ TeV		
$M_h^{\text {old}}$ (GeV)	128.63	128.63	128.63
$M_h^{\text {new}}$ (GeV)	129.62	129.61	129.59
$m_h^{\text {mod}-}$-like	$M_{\text {SUSY}}=2$ TeV		
$M_h^{\text {old}}$ (GeV)	126.92	126.92	126.92
$M_h^{\text {new}}$ (GeV)	127.34	127.33	127.44
	$M_{\text {SUSY}}=3$ TeV		
$M_h^{\text {old}}$ (GeV)	127.02	127.02	127.02
$M_h^{\text {new}}$ (GeV)	127.80	127.80	127.94

The differences in the renormalization schemes and the dependence on the parameters in the stop sector are further investigated in Table 2. Here the shifts in the light Higgs-boson mass are shown for SUSY scales of 2 and 3 TeV, using otherwise the parameters of the $m_h^{\text {mod}+}$ and $m_h^{\text {mod}-}$ scenarios. The results for $M_h^{\text {old}}+\mathcal {O}(\alpha _t\alpha _s p^2)+\mathcal {O}(\alpha \alpha _s)$ from Ref. [54], where the mass and Yukawa coupling of the bottom quark have been neglected, are labelled as $M_h^{\text {new}}$ in Table 2. Two versions of the results from Ref. [54] are shown, one using the renormalization scheme adopted in Ref. [54] with $\delta ^{(2)}Z_{\mathcal {H}_{i}}=\delta ^{(2)}Z_{\mathcal {H}_{i}}^{{\text {Ref. }[54]}}$, and the other using the renormalization scheme of Ref. [53], which we have adopted in the present work, with $\delta ^{(2)}Z_{\mathcal {H}_{i}}=\delta ^{(2)}Z_{\mathcal {H}_{i}}^{{\text {Ref. }[53]}}$.^6^ It can be seen in Table 2 that there is very good agreement, at the level of about 10 MeV, between our results and the results from Ref. [54] using the renormalization scheme of Ref. [53]. The different choices of renormalization schemes in the result of Ref. [54] amount to mass shifts of up to 150 MeV for the displayed examples. The difference between $M_h^{\text {new}}$ and $M_h^{\text {old}}$ increases with $M_{\text {SUSY}}$ and reaches up to 1 GeV for the $m_h^{\text {mod}+}$-like scenario at 3 TeV.

### Scenario 1: $m_h^{\text {mod}}$-like

In the following we further investigate the numerical impact of our results, including the effect of non-zero phases of the complex parameters. We start with an $m_h^{\text {mod}}$-like scenario. The MSSM model parameters in this scenario are chosen as follows

5.3 $$\begin{aligned} m_{H^\pm }&= 1.5\,\text {TeV}, \quad M_2 = 500\,\text {GeV}, \quad |M_3|= 2.5\,\text {TeV},\nonumber \\ m_{\{\tilde{t},\tilde{b}\}_{\text {L}}}&= m_{\tilde{Q}_3} = 2.1\,\text {TeV}, \quad m_{\{\tilde{t},\tilde{b}\}_{\text {R}}} = 2\,\text {TeV}, \nonumber \\ |X_t|&= 1.3\,m_{\tilde{t}_{\text {R}}}, |A_b|= |A_t|,\nonumber \\ m_{\{ \tilde{q},\tilde{l} \}_{\{ \text {L},\text {R} \}}}&= 2.5 \,\text {TeV}, \quad A_{\{q,l\}} = 0,\nonumber \\&\quad q \in {u,d,s,c},\quad l \in {e,\mu ,\tau }. \end{aligned}$$

Compared to the original $m_h^{\text {mod}}$ scenario we choose larger bilinear soft-breaking parameters for the sfermions, and also larger absolute values for $\mu$ (see below) and $M_2$. Thereby $m_{\tilde{Q}_3}$ is slightly different from $m_{\{\tilde{t},\tilde{b}\}_{\text {R}}}$ in order to avoid numerical instabilities by degeneracies. However, the general feature of this scenario is kept: it allows for a wide range of $X_t = A_t^* - \mu /\tan \beta$ to be in agreement with experimental bounds. With our choice of parameters, $A_t$ and $A_b$ are not expected to be affected by constraints from charge- and color-breaking minima [132–139]. As $A_\tau$ has negligible impact on the Higgs mass prediction, we set it to zero.

**Fig. 7:**
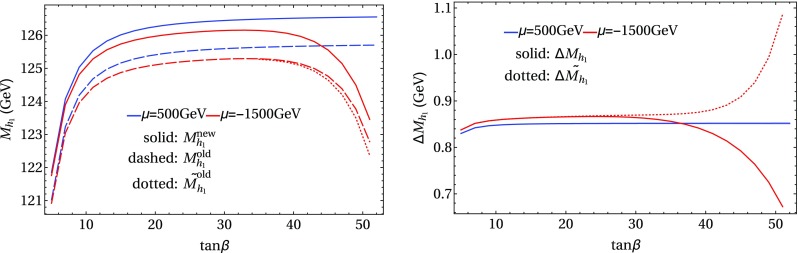
Prediction for the light Higgs-boson mass $M_{h_1}$ (left) and the mass shifts $\Delta M_{h_1}$, $\Delta \tilde{M}_{h_1}$ [right, as defined in Eq. (5.2)] as a function of $\text {tan}\beta$ using $m_A$ as input mass for different values of $\mu$. Parameters are as described in (5.3)

**Fig. 8:**
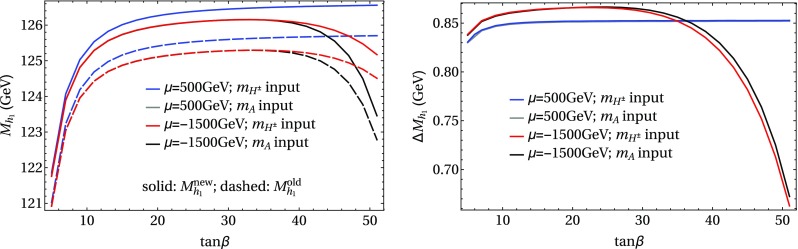
Prediction for the light Higgs-boson mass ${M}_{h_1}$ (left) and the mass shifts $\Delta M_{h_1}$, $\Delta \tilde{M}_{h_1}$ [right, as defined in Eq. (5.2)] as a function of $\text {tan}\beta$ using $m_{H^\pm }$ as input mass for different values of $\mu$. The black lines show the results of Fig. 7 for $\mu =-1500$ GeV. The results of Fig. 7 for $\mu =500$ GeV are indicated by grey lines, which are underneath the blue lines. Parameters are as described in Eq. (5.3)

First, the dependence of the lightest Higgs-boson mass $M_{h_1}$ on $\tan \beta$ is analyzed for different values of the $\mu$ parameter. Setting all phases of the parameters that can be complex to zero, our result can be compared to previous ones in the MSSM with real parameters where the corrections evaluated in the present paper were not included. In the considered scenario, it is possible to choose either $m_A$ or $m_{H^\pm }=\sqrt{m_A^2 + M_W^2}$ as an input parameter which is renormalized on-shell accordingly. The chosen input mass for Fig. 7 is $m_A$. A comparison of the predicted mass from FeynHiggs-2.12.0, with ($M_{h_1}^{\text {new}}$) and without ($M_{h_1}^{\text {old}}$) incorporating our new corrections is shown. Solid lines depict the new, dashed lines the previous results. In order to illustrate the different relative sizes of our new contributions, we further plot $\tilde{M}_{h_1}^{\text {old}}$, where the FeynHiggs result for $M_{h_1}^{\text {old}}$ is supplemented with the $\mathcal {O}{\left( \alpha _b\alpha _s\right) }$ terms known in the MSSM with real parameters (dotted lines). The prediction with $\mu =500$ GeV is shown in blue, while the resulting Higgs-boson mass using $\mu =-1500$ GeV is shown in red. The blue dashed and blue dotted lines are lying on top of each other which means that the $\mathcal {O}{\left( \alpha _b\alpha _s\right) }$ corrections are negligible in this case. The red curves show that our new corrections are significantly larger than the pure $\mathcal {O}{\left( \alpha _b\alpha _s\right) }$ contributions and enter with different sign. They therefore overcompensate the slight downward shift induced by the pure $\mathcal {O}{\left( \alpha _b\alpha _s\right) }$ contributions. The differences $\Delta M_{h_1}$ and $\Delta \tilde{M}_{h_1}$, as defined in Eq. (5.2), are plotted on the right-hand side of Fig. 7. For low values of $\text {tan}\beta$ the new corrections slightly increase and then stay constant over a wide range. Only for values $\tan \beta >40$ and large negative $\mu$ they drop by about $20\%$. Values for $\tan \beta$ above the depicted range and large negative $\mu$ lead to a further rapid decrease of $M_{h_1}$, eventually yielding a tachyonic Higgs boson. This is due to the large bottom Yukawa coupling with resummed $\tan \beta$-enhanced terms which can become non-perturbative in that region of the parameter space. The rise of the red dotted curve at large $\tan \beta$ reflects that this decrease happens for larger values of $\tan \beta$ once our new corrections are taken into account.

In Fig. 8 the charged Higgs mass $m_{H^\pm }$ is used as an input parameter. The latter implies the occurrence of terms of $\mathcal {O}{\left( \sqrt{\alpha _q}\sqrt{\alpha _o}\alpha _s\right) }$ and corresponds to the renormalization scheme compatible with both the MSSM with real and complex parameters. On the left-hand side of Fig. 8, the blue ($\mu =500$ GeV) and red ($\mu =-1500$ GeV) lines show the prediction for the lightest Higgs mass with (solid) and without (dashed) our new contributions. In addition, the solid and dashed curves of Fig. 7 are indicated again as grey ($\mu =500$ GeV) and black ($\mu =-1500$ GeV) lines. In this way, the influence of the two different renormalization schemes on the Higgs-mass prediction can be seen. While the blue and grey lines lie on top of each other over the whole range of $\tan \beta$, deviations of up to 1.5 GeV can be observed between the red and black curves in the region of large $\tan \beta$. Since the slope of the red curves for large $\tan \beta$ is smaller than for the black curves, the renormalization scheme with $m_{H^\pm }$ as input parameter is better suited for this particular region in parameter space. On the right-hand side of Fig. 8 the mass shifts $\Delta M_{h_1}$ and $\Delta \tilde{M}_{h_1}$ resulting from our new contributions are depicted. The color coding is the same as described before. The size of the shifts is almost invariant under the exchange of $m_A$ and $m_{H^\pm }$ as input parameter, since only small differences between the two renormalization schemes can be noticed.

We note that setting $\mu =1500$ GeV and using $m_{H^\pm }$ as input, the same qualitative behavior as for the lower positive $\mu$ value can be observed, with the new contributions being of the same size as for $\mu =-1500$ GeV in the low and intermediate $\tan \beta$ region. Furthermore, the size of the mass shift $\Delta M_{h_1}$ in Figs. 7 and 8 shows a similar tendency with respect to the chosen sfermion masses as depicted in Table 2, i.e. larger scales increase the size of the new corrections. However, for stop- and sbottom masses larger than $\approx 2$ TeV logarithmic contributions of higher order also become important. Then, a resummation of these logarithms should be taken into account for an accurate Higgs-mass prediction. The gluino mass can have a sizable impact due to its appearance in the threshold correction of $\mathcal {O}{\left( \alpha _t\alpha _s\right) }$.^7^

### Scenario 2: large $\tan \beta$

Scenarios with large values of $\tan \beta$ are particularly interesting for investigating effects of the new contributions in the bottom and sbottom sector. In that parameter region, terms proportional to the bottom Yukawa coupling can be as important as terms from the top sector. In the following, we investigate the dependence of the new contributions on various parameters at a fixed large $\tan \beta$ value. In order to be consistent with experimental constraints by ATLAS and CMS we choose a sufficiently large value of $m_{H^\pm }$ [140, 141]. If not stated otherwise, the MSSM model parameters are

5.4 $$\begin{aligned} \tan \beta&= 50, \quad \mu = -1.5\,\text {TeV}, \quad m_{H^\pm } = 1.5\,\text {TeV}, \nonumber \\ M_2&= 500\,\text {GeV}, \quad |M_3| = 2.5\,\text {TeV},\nonumber \\ m_{\{\tilde{t},\tilde{b}\}_{\text {L}}}&= m_{\tilde{Q}_3} = 2.1\,\text {TeV}, \quad m_{\{\tilde{t},\tilde{b}\}_{\text {R}}} = 2\,\text {TeV}, \nonumber \\ |X_t|&= 1.3\,m_{\tilde{b}_{\text {R}}}, \quad |A_b|= |A_t|,\nonumber \\ m_{\{ \tilde{q},\tilde{l} \}_{\{ \text {L},\text {R} \}}}&= 2.5 \,\text {TeV}, \quad A_{\{q,l\}} = 0,\nonumber \\&\quad q \in {u,d,s,c}, \quad l \in {e,\mu ,\tau } . \end{aligned}$$

**Fig. 9:**
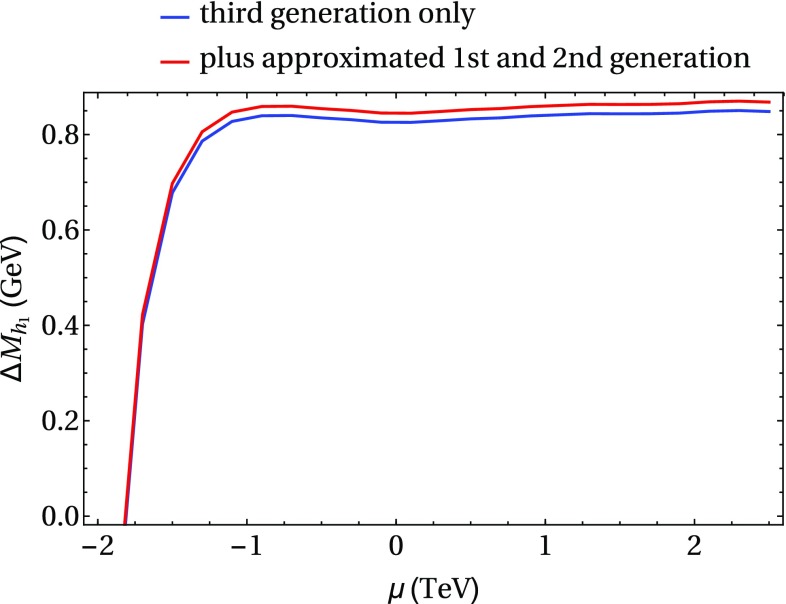
Variation of the mass shift $\Delta M_{h_1}$ with $\mu$. The blue curve shows the result including contributions only from the 3rd generation. The red line shows the result where also contributions of the 1st and 2nd generation are included using the approximation $m_q=0,\,q\in \{c,s,u,d\}$. Parameters are as described in Eq. (5.4)

In Fig. 9 the mass shift $\Delta M_{h_1}$ is displayed as a function of $\mu$. Over a wide range the mass shift is nearly constant at about $\Delta M_{h_1}\approx 0.85$ GeV. Only for large negative values $\mu \lesssim -1.8$ TeV, the correction to the lightest Higgs falls steeply indicating a parameter region where the perturbative prediction for $M_{h_1}$ becomes unreliable owing to the large value of the bottom Yukawa coupling. Thus, $\mu$ should be kept above that value. The blue line shows the effect of only the third generation quarks and squarks in our new contributions. The red line shows the result where these contributions are supplemented with the corrections of the first and second generation, neglecting the light quark masses and Yukawa couplings of the first two generations, $m_q=0,\,q\in \{c,s,u,d\}$. Accordingly, the difference between the two curves is given by the pure gauge contributions of $\mathcal {O}{\left( \alpha \alpha _s\right) }$ from the first and second generation. They are rather small, amounting to about 30 MeV.

**Fig. 10:**
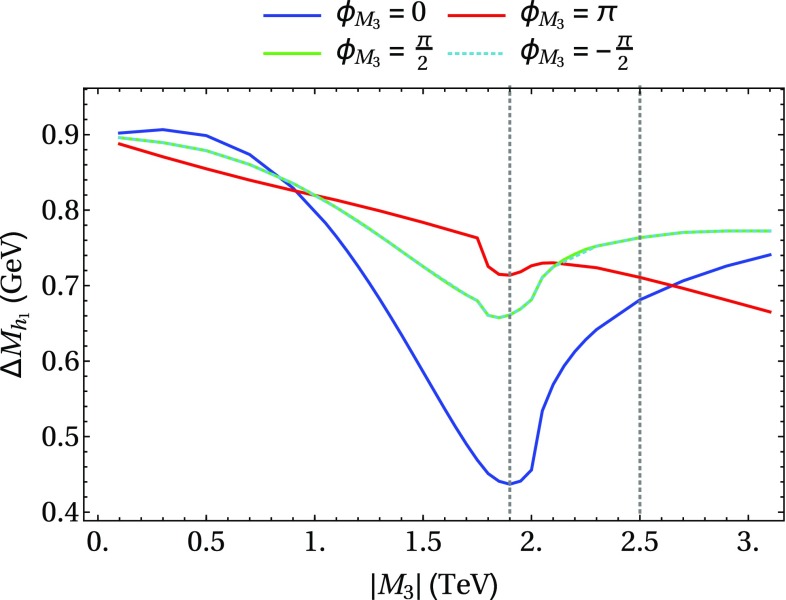
Variation of the mass shift $\Delta M_{h_1}$ with the absolute value and phase of the gluino mass parameter $M_3 = |M_3|\exp {\left( {i}\,\phi _{M_3}\right) }$. The vertical dashed lines are at $|M_3|= 1900$ and 2500 GeV. The dependence on $\phi _{M_3}$ at those values of $|M_3|$ is illustrated in Fig. 11. Parameters are as described in Eq. (5.4)

**Fig. 11:**
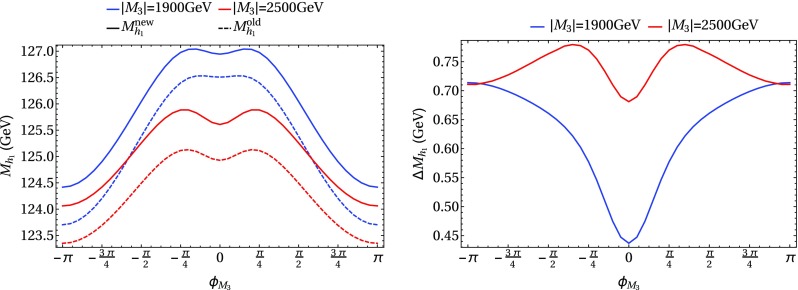
Variation of the light Higgs-boson mass $M_{h_1}$ (left) and the mass shift $\Delta M_{h_1}$ (right) with the gluino phase $\phi _{M_3}$, while all other phases are set to zero. Parameters are as described in Eq. (5.4)

**Fig. 12:**
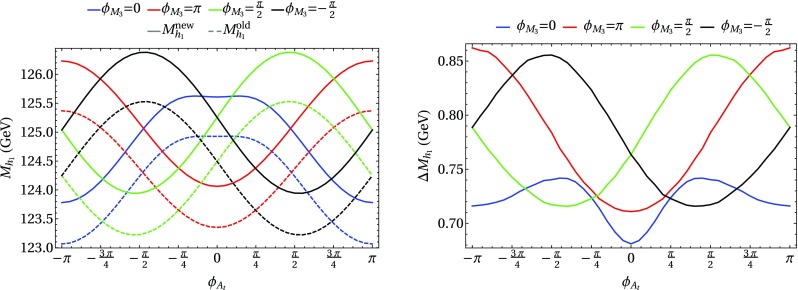
Variation of the light Higgs-boson mass $M_{h_1}$ (left) and the mass shift $\Delta M_{h_1}$ (right) with the phase $\phi _{A_t}$ for different $\phi _{M_3}$ and $\phi _{A_b}=0$. Parameters are as described in Eq. (5.4)

The variation of $\Delta M_{h_1}$ with the gluino-mass parameter $M_3 = |M_3| \exp {\left( {i}\,\phi _{M_3}\right) }$ is shown in Fig. 10. Close to $|M_3|\approx 1.9$ TeV, thresholds of the gluino–fermion–sfermion system can be observed, which are introduced by one-loop integrals entering via the subloop-renormalization and resummation of the bottom Yukawa coupling. The effect of varying the absolute value of the gluino-mass parameter $|M_3|$ on $\Delta M_{h_1}$ is strongest for $\phi _{M_3} = 0$ and successively weakened as $\phi _{M_3}$ approaches $\pi$. The results for $\phi _{M_3} = \pm \frac{\pi }{2}$ almost lie on top of each other.

**Fig. 13:**
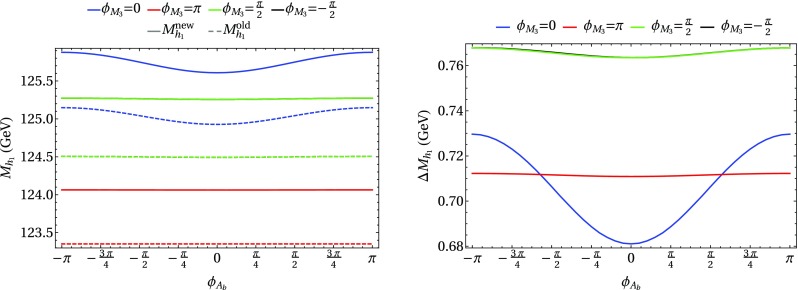
Variation of the light Higgs-boson mass $M_{h_1}$ (left) and the mass shift $\Delta M_{h_1}$ (right) with the phase $\phi _{A_b}$ for different $\phi _{M_3}$ and $\phi _{A_t}=0$. The results for $\phi _{M_3}=\pm \frac{\pi }{2}$ lie on top of each other. Parameters are as described in Eq. (5.4)

In Figs. 11, 12 and 13 the dependence on the three phases $\phi _{M_3},\,\phi _{A_t}$ and $\phi _{A_b}$ is displayed, respectively. The impact of the new (solid) corrections in comparison with the ones implemented so far in FeynHiggs (dashed) are shown for the lightest Higgs-boson mass on the left-hand side of each figure, while the differences $\Delta M_{h_1}$ are shown on the right-hand side. Comparing to the MSSM with real parameters, where the phases are equal to zero or $\pi$, sizable differences for the prediction of the lightest Higgs-boson mass are visible. Concerning the total variation of $M_{h_1}$ including all now available corrections, the impact of the phases $\phi _{A_t}$ and $\phi _{M_3}$ is seen to be rather large with effects that can exceed 2 GeV, while varying the phase $\phi _{A_b}$ yields only rather small shifts of $\approx 0.2\,\text {GeV}$.

The prediction for $M_{h_1}$ as function of $\phi _{M_3}$ shown in Fig. 11 is symmetric with respect to the sign of $\phi _{M_3}$. The variation of $\Delta M_{h_1}$ with $\phi _{M_3}$ is shown on the right-hand side of Fig. 11. The pronounced dependence on the absolute value of $|M_3|$ seen in Fig. 10 can be observed again. The variation of $\phi _{M_3}$ changes $\Delta M_{h_1}$ by up to 250 MeV for an $|M_3|$ value around the gluino–fermion–sfermion threshold, while for $|M_3|=2.5$ GeV $\Delta M_{h_1}$ is shifted only by up to 70 MeV.

The phase dependence of $\Delta M_{h_1}$ on $\phi _{A_t}$ and $\phi _{A_b}$ is shown on the right-hand side of Figs. 12 and 13, respectively. The variation of $\Delta M_{h_1}$ with $\phi _{A_t}$ and $\phi _{A_b}$ is seen to be rather small. It reaches up to 150 MeV for the phase $\phi _{A_t}$ and up to 50 MeV for $\phi _{A_b}$. It should be noted that the results for $\phi _{M_3} = \pm \frac{\pi }{2}$ lie on top of each other in Fig. 13. While the variation with $\phi _{A_b}$ is rather small for any non-zero $\phi _{M_3}$, the variation with $\phi _{A_t}$ is minimal for $\phi _{M_3}=0$ and maximal for $\phi _{M_3}=\pi$. Using different values of $\phi _{A_b}$ (and keeping $\phi _{M_3}$ fixed) has only a small effect on the variation of $\Delta M_{h_1}$ with $\phi _{A_t}$. The corresponding plot is therefore not shown here.

### Scenario 3: low $M_H$

In the low-$M_H$ scenario the observed SM-like Higgs boson with a mass of about 125 GeV can be identified with the next-to-lightest neutral *CP*-even Higgs boson of the MSSM, see Ref. [5] for a recent update. We choose the following MSSM model parameters,

5.5 $$\begin{aligned} \tan \beta&= 6.5, \quad \mu = 5\,\text {TeV}, \nonumber \\ M_2&= 300\,\text {GeV}, \quad |M_3|= 1.5\,\text {TeV},\nonumber \\ m_{\{\tilde{t},\tilde{b}\}_{\{\text {L},\text {R}\}}}&= 750\,\text {GeV}, \quad m_{\tilde{\tau }_{\{\text {L},\text {R}\}}} = 500\,\text {GeV},\nonumber \\ m_{\tilde{q}_{\{ \text {L},\text {R} \}}}&= 1.5\,\text {TeV}, \quad m_{\tilde{l}_{\{\text {L},\text {R}\}}} = 250\,\text {GeV},\nonumber \\ A_t = A_b = A_{\tau }&= -70\,\text {GeV}, \quad A_{\{q,l\}} = 0,\,\, q \in {u,d,s,c}, \nonumber \\&\quad l \in {e,\mu }. \end{aligned}$$

Compared to the original scenario in [5] we had to choose a smaller value of $\mu$ in order to avoid a tachyonic lightest Higgs boson for a charged Higgs mass $m_{H^\pm }\approx 160$ GeV. Our value for $\tan \beta$ is chosen such that the scenario is valid according to Fig. 26 of [5].

**Fig. 14:**
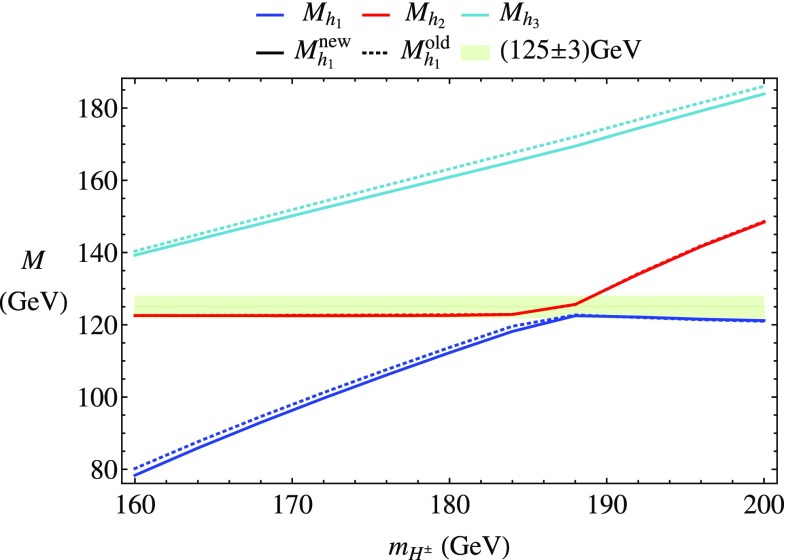
Variation of the three neutral Higgs-boson masses $M_{h_i}$ with the charged Higgs boson mass $m_{H^\pm }$. The results for $M_{h_i}^{\text {new}}$ are shown as full lines and those for $M_{h_i}^{\text {old}}$ as dotted lines. Parameters are as described in Eq. (5.5)

In Fig. 14 the three neutral Higgs-boson masses are depicted, varying the charged Higgs-boson mass $m_{H^\pm }$ which is used as an input parameter. The light green band illustrates the mass range of $125\pm 3$ GeV; it should be interpreted as a rough indication of the mass range which is theoretically in agreement with the detected Higgs boson. Up to $m_{H^\pm }\lesssim 188$ GeV the heavier Higgs $h_2$ could be associated with the discovered Higgs-like particle; however, as can be seen in the low-$M_H^{\text {alt}+}$ scenario in Fig. 26 of [5], our choice of $\mu$ and $\tan \beta$ is already excluded for a charged Higgs mass $m_{H^\pm }=185$ GeV. Yet, scenarios with values of $m_{H^\pm }$ closer to or below $m_t$ are still allowed. In this region the new corrections presented here have a negligible impact on $M_{h_2}$, but lead to a downward shift of about 1 GeV for both $M_{h_1}$ and $M_{h_3}$.

**Fig. 15:**
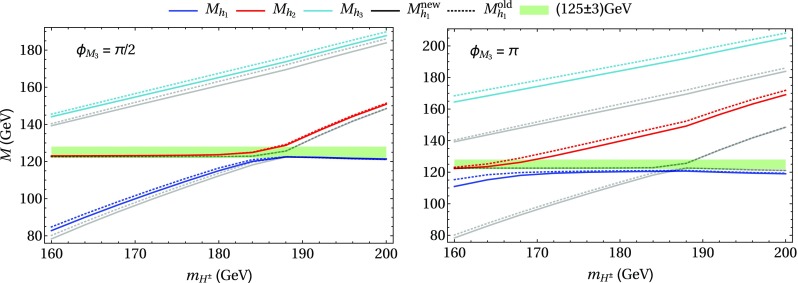
Variation of the three neutral Higgs-boson masses $M_{h_i}$ with the charged Higgs boson mass $m_{H^\pm }$ for non-zero phases $\phi _{M_3}$. The results for $M_{h_i}^{\text {new}}$ are shown as full lines and those for $M_{h_i}^{\text {old}}$ as dotted lines. The results of Fig. 14 with $\phi _{M_3} = 0$ are depicted in grey for reference. Parameters are as described in Eq. (5.5)

As shown in Fig. 15, using a non-zero value of the gluino phase of $\phi _{M_3} = \pi /2$ or $\phi _{M_3} = \pi$ shifts all three neutral Higgs masses to larger values as compared to the case $\phi _{M_3}=0$. For better comparison, the results of Fig. 14 are underlaid in grey. The numerical impact of the new contributions presented here rises with increasing $\phi _{M_3}$. For $\phi _{M_3}=\pi$ all neutral Higgs masses can receive large corrections of up to 5 GeV.

## Conclusions

We have computed the full two-loop QCD corrections to the lightest Higgs-boson mass in the MSSM with complex parameters. Compared to previous works, this primarily involves going beyond the gaugeless limit, and including a finite bottom-quark mass; furthermore the momentum dependence of loop integrals is taken into account. On the technical side, this involves the computation of 177 different mass topologies evaluated at different kinematical configurations, amounting to a total of 513 two-point two-loop integrals with up to five mass scales. These integrals have been computed numerically with the program SecDec.

In the first part of our numerical analysis, we have compared our results with earlier result in the literature taking the appropriate limit of real parameters and/or vanishing external momentum of our results. We have found very good agreement with the existing results in the appropriate limit if the same renormalization scheme is employed. The contributions evaluated in this paper yield a shift in the lightest Higgs-boson mass at the level of 1 GeV, where the impact has been seen to be more pronounced for an increasing mass scale of the stops.

We have furthermore investigated the dependence of the new corrections on $\tan \beta$ choosing different values of the $\mu$-parameter as well as different renormalization schemes. For a large negative $\mu$ the corrections are generally larger and amount to around 0.9 GeV in $M_{h_1}$. The corrections are largest for $10<\tan \beta <30$, decrease by $3\%$ for lower values and by about $20\%$ beyond $\tan \beta =30$.

We find non-vanishing mixed up- and down-type Yukawa corrections in the charged Higgs-boson self-energy correction entering the mass predictions for the neutral Higgs bosons as renormalization constant if the charged Higgs mass $m_{H^\pm }$ instead of the neutral $\mathcal {CP}$-odd mass $m_A$ is chosen as an input parameter. We have compared the mass prediction for the lightest Higgs boson in both schemes and have found good agreement in general. However, using the charged Higgs mass as an input parameter yields better numerical stability at large $\tan \beta$ and large negative $\mu$.

The Yukawa contributions scale according to their Yukawa couplings, leading to much smaller contributions from the first and second generation quarks and squarks. The pure gauge terms of $\mathcal {O}{\left( \alpha \alpha _s\right) }$ in the limit of massless quarks are found to be of similar small size, below 20 MeV for one generation.

Analyzing the dependence on the gluino mass, we have found maximal shifts of $\approx 900$ MeV in $M_{h_1}$. The corrections show a sensitive dependence on the gluino–fermion–sfermion threshold, which enters via the counterterms of our renormalization scheme, and the gluino phase. For the $\mu$-parameter a mass shift of the lightest Higgs by $\approx 850$ MeV is found over large regions of parameter space.

Concerning the impact of the three phases $\phi _{M_3}$, $\phi _{A_t}$ and $\phi _{A_b}$, we find significant effects in our new corrections from varying the gluino phase and the pase of $A_t$. For $\phi _{M_3}$ the phase dependence becomes particularly pronounced in the threshold region of the gluino–fermion–sfermion system, as mentioned above.

Besides scenarios where the lightest neutral Higgs boson in the spectrum of the MSSM is the SM-like state that can be identified with the detected Higgs signal, we have also analyzed the impact of the newly computed contributions on the Higgs-mass predictions for the three neutral Higgs bosons within the low-$M_H$ scenario for different values of the gluino phase $\phi _{M_3}$. We have found mass corrections of $\approx 1$ GeV for $\phi _{M_3}=0$ and up to $\approx 5$ GeV for $\phi _{M_3}=\pi$ in this case.

Accordingly, we have found that the subleading two-loop contributions that we have evaluated in this paper yield a shift in the prediction for the mass of the light SM-like Higgs boson of the MSSM of up to the level of 1 GeV. The size of the correction sensitively depends on the mass scales of the stops and sbottoms, on the absolute value and phase of the gluino mass parameter, as well as on the absolute value and phase of the trilinear coupling in the stop sector (and to a lesser extent on the trilinear coupling in the sbottom sector). While these findings of course have an impact on the remaining theoretical uncertainties from unknown higher-order corrections, we do not attempt to provide an improved estimate of the remaining uncertainties here. Such an improved estimate should be based on a combination of the fixed-order result considered here with a resummation of higher-order logarithmic contributions. We leave such an analysis to future work.

It should be noted in this context that our results for the corrections of $\mathcal {O}{\left( \alpha \alpha _s\right) }$ beyond the gaugeless limit cannot be used directly to infer the possible size of the corresponding contributions of $\mathcal {O}{\left( \alpha ^2\right) }$ to the Higgs-boson spectrum, which are unknown up to now. This is due to the fact that the requirement of a strong coupling in the corrections that we have evaluated significantly constrains the structure of the contributing Feynman diagrams, while additional classes of contributions will have to be taken into account for a full calculation of the corrections of $\mathcal {O}{\left( \alpha ^2\right) }$.

The new contributions evaluated in this paper will be made publicly available in the program FeynHiggs.

## Footnotes

1 Within the MSSM it is usually assumed that the observed particle is associated with the lightest neutral Higgs boson of the model; see Ref. [5] for a recent update on the viability of the interpretation in terms of the next-to-lightest neutral Higgs boson of the MSSM.

2 We use a lower-case *m* for the Higgs-boson masses at the tree level.

3 The Goldstone bosons can acquire a non-zero mass value by gauge fixing.

4 We use an uppercase *M* for the Higgs masses at higher order.

5 These one-loop functions are given by

$$\begin{aligned} B_{1}{\left( 0, m_1, m_2\right) }&= -\frac{1}{2} B_{0}{\left( 0, m_1, m_2\right) } + \frac{m_2 - m_1}{2} DB_0{\left( 0, m_1, m_2\right) },\\ B_{0}{\left( 0, m_1, m_2\right) }&= \frac{A_0{\left( m_1\right) } - A_0{\left( m_2\right) }}{m_1 - m_2}\ ,\\ DB_0{\left( 0, m_1, m_2\right) }&= \frac{m_1^2 - m_2^2 + 2\, m_1\, m_2 \log {\tfrac{m_2}{m_1}}}{2\, (m_1 - m_2)^3},\\ A_0{\left( m\right) }&= m \left( 1 - \log {\tfrac{m}{\mu _{r}}}\right) . \end{aligned}$$

6 We are very grateful to S. de Vita for providing us with those results.

7 In the scenario of Eq. (5.3) the NNLL-resummation of logarithms with stop masses as implemented in FeynHiggs gives rise to an upward shift of the lightest Higgs mass $M_h^{\text {old}}$ by $\approx 1.5$ GeV over the whole range of $\tan \beta$; for stop-mass scales at 3 TeV this shift already amounts to $\approx 2.7$ GeV. Changing the gluino mass from 2.5 to 4 TeV corresponds to a downward shift of the SM-like Higgs mass due to the threshold corrections of $\mathcal {O}{\left( \alpha _t\alpha _s\right) }$ of $\approx 0.5$ GeV over the whole range of $\tan \beta$.

## References

[1] ATLAS Collaboration, Phys. Lett. B **716**, 1 (2012). arXiv:1207.7214

[2] CMS Collaboration, Phys. Lett. B **716**, 30 (2012). arXiv:1207.7235

[3] ATLAS Collaboration, CMS Collaboration, D. Sperka, 53rd Rencontres de Moriond, March 2018, La Thuile, Italy. Measurements of the BEH scalar mass and other couplings in ATLAS and CMS

[4] ATLAS Collaboration, CMS Collaboration, JHEP **08**, 045 (2016). arXiv:1606.02266

[5] Bechtle P, Haber H, Heinemeyer S, Stål O, Stefaniak T, Weiglein G, Zeune L (2017). Eur. Phys. J. C.

[6] Pilaftsis A (1998). Phys. Rev. D.

[7] Demir D (1999). Phys. Rev. D.

[8] Pilaftsis A, Wagner C (1999). Nucl. Phys. B.

[9] Heinemeyer S (2001). Eur. Phys. J. C.

[10] Frank M, Hahn T, Heinemeyer S, Hollik W, Rzehak H, Weiglein G (2007). JHEP.

[11] Degrassi G, Heinemeyer S, Hollik W, Slavich P, Weiglein G (2003). Eur. Phys. J. C.

[12] Heinemeyer S, Hollik W, Weiglein G (1999). Eur. Phys. J. C.

[13] Heinemeyer S, Hollik W, Weiglein G (1998). Phys. Rev. D.

[14] Heinemeyer S, Hollik W, Weiglein G (1999). Phys. Lett. B.

[15] Haber H, Hempfling R (1991). Phys. Rev. Lett..

[16] Ellis J, Ridolfi G, Zwirner F (1991). Phys. Lett. B.

[17] Okada Y, Yamaguchi M, Yanagida T (1991). Prog. Theor. Phys..

[18] Okada Y, Yamaguchi M, Yanagida T (1991). Phys. Lett. B.

[19] Ellis J, Ridolfi G, Zwirner F (1991). Phys. Lett. B.

[20] Sasaki K, Carena M, Wagner C (1992). Nucl. Phys. B.

[21] Chankowski P, Pokorski S, Rosiek J (1992). Phys. Lett. B.

[22] Brignole A (1992). Phys. Lett. B.

[23] Hempfling R, Hoang A (1994). Phys. Lett. B.

[24] Casas J, Espinosa J, Quiros M, Riotto A (1995). Nucl. Phys. B.

[25] Dabelstein A (1995). Z. Phys. C.

[26] Carena M, Espinosa J, Quiros M, Wagner C (1995). Phys. Lett. B.

[27] Carena M, Quiros M, Wagner C (1996). Nucl. Phys. B.

[28] Pierce D, Bagger J, Matchev K, Zhang R (1997). Nucl. Phys. B.

[29] Haber H, Hempfling R, Hoang A (1997). Z. Phys. C.

[30] Heinemeyer S, Hollik W, Weiglein G (1998). Phys. Lett. B.

[31] Zhang R (1999). Phys. Lett. B.

[32] Espinosa J, Zhang R (2000). JHEP.

[33] Carena M, Haber H, Heinemeyer S, Hollik W, Wagner C, Weiglein G (2000). Nucl. Phys. B.

[34] Espinosa J, Zhang R (2000). Nucl. Phys. B.

[35] Espinosa J, Navarro I (2001). Nucl. Phys. B.

[36] Degrassi G, Slavich P, Zwirner F (2001). Nucl. Phys. B.

[37] Martin S (2002). Phys. Rev. D.

[38] Brignole A, Degrassi G, Slavich P, Zwirner F (2002). Nucl. Phys. B.

[39] Dedes A, Slavich P (2003). Nucl. Phys. B.

[40] Martin S (2002). Phys. Rev. D.

[41] Dedes A, Degrassi G, Slavich P (2003). Nucl. Phys. B.

[42] Heinemeyer S, Hollik W, Rzehak H, Weiglein G (2005). Eur. Phys. J. C.

[43] Martin S (2003). Phys. Rev. D.

[44] Martin S (2004). Phys. Rev. D.

[45] Allanach B, Djouadi A, Kneur J, Porod W, Slavich P (2004). JHEP.

[46] Heinemeyer S, Hollik W, Weiglein G (2006). Phys. Rep..

[47] Martin S (2005). Phys. Rev. D.

[48] Martin S, Robertson D (2006). Comput. Phys. Commun..

[49] Harlander R, Kant P, Mihaila L, Steinhauser M (2008). Phys. Rev. Lett..

[50] Kant P, Harlander R, Mihaila L, Steinhauser M (2010). JHEP.

[51] Brignole A, Degrassi G, Slavich P, Zwirner F (2002). Nucl. Phys. B.

[52] Harlander R, Klappert J, Voigt A (2017). Eur. Phys. J. C.

[53] Borowka S, Hahn T, Heinemeyer S, Heinrich G, Hollik W (2014). Eur. Phys. J. C.

[54] Degrassi G, Di Vita S, Slavich P (2015). Eur. Phys. J. C.

[55] Borowka S, Hahn T, Heinemeyer S, Heinrich G, Hollik W (2015). Eur. Phys. J. C.

[56] Draper P, Lee G, Wagner C (2014). Phys. Rev. D.

[57] Pardo Vega J, Villadoro G (2015). JHEP.

[58] G. Lee, C. Wagner, (2015). arXiv:1508.00576

[59] Hahn T, Heinemeyer S, Hollik W, Rzehak H, Weiglein G (2014). Phys. Rev. Lett..

[60] Bahl H, Hollik W (2016). Eur. Phys. J. C.

[61] H. Bahl, S. Heinemeyer, W. Hollik, G. Weiglein, (2017). arXiv:1706.00346 10.1103/PhysRevLett.112.141801 24765944

[62] P. Athron, J. Park, T. Steudtner, D. Stöckinger, A. Voigt, (2016). arXiv:1609.00371

[63] Bagnaschi E, Giudice GF, Slavich P, Strumia A (2014). JHEP.

[64] Bagnaschi E, Pardo Vega J, Slavich P (2017). Eur. Phys. J. C.

[65] Martin S (2003). Phys. Rev. D.

[66] Martin S (2005). Phys. Rev. D.

[67] Martin S (2007). Phys. Rev. D.

[68] Choi S, Drees M, Lee J (2000). Phys. Lett. B.

[69] Ibrahim T, Nath P (2001). Phys. Rev. D.

[70] Ibrahim T, Nath P (2002). Phys. Rev. D.

[71] Carena M, Ellis J, Pilaftsis A, Wagner C (2000). Nucl. Phys. B.

[72] Heinemeyer S, Hollik W, Rzehak H, Weiglein G (2007). Phys. Lett. B.

[73] Hollik W, Paßehr S (2014). Phys. Lett. B.

[74] Hollik W, Paßehr S (2014). JHEP.

[75] S. Paßehr, G. Weiglein, (2017). arXiv:1705.07909

[76] Goodsell M, Staub F (2017). Eur. Phys. J. C.

[77] Liebler S, Patel S, Weiglein G (2017). Eur. Phys. J. C.

[78] Heinemeyer S, Hollik W, Weiglein G (2000). Comput. Phys. Commun..

[79] Lee J, Pilaftsis A, Carena M, Choi S, Drees M, Ellis J, Wagner C (2004). Comput. Phys. Commun..

[80] Williams K, Rzehak H, Weiglein G (2011). Eur. Phys. J. C.

[81] Collaboration ACME (2014). Science.

[82] Pospelov M, Ritz A (2005). Ann. Phys..

[83] Pendlebury J (2015). Phys. Rev. D.

[84] Baker C (2006). Phys. Rev. Lett..

[85] Baker C (2007). Phys. Rev. Lett..

[86] Serebrov A (2015). Phys. Rev. C.

[87] Graner B, Chen Y, Lindahl E, Heckel B (2016). Phys. Rev. Lett..

[88] Yamanaka N, Sahoo B, Yoshinaga N, Sato T, Asahi K, Das B (2017). Eur. Phys. J. A.

[89] Drechsel P, Galeta L, Heinemeyer S, Weiglein G (2017). Eur. Phys. J. C.

[90] Drechsel P, Gröber R, Heinemeyer S, Mühlleitner M, Rzehak H, Weiglein G (2017). Eur. Phys. J. C.

[91] Domingo F, Drechsel P, Paßehr S (2017). Eur. Phys. J. C.

[92] Weiglein G, Scharf R, Böhm M (1994). Nucl. Phys. B.

[93] Hahn T, Paßehr S (2017). Comput. Phys. Commun..

[94] Carter J, Heinrich G (2011). Comput. Phys. Commun..

[95] Borowka S, Carter J, Heinrich G (2013). Comput. Phys. Commun..

[96] Borowka S, Heinrich G (2013). Comput. Phys. Commun..

[97] Hahn T, Heinemeyer S, Hollik W, Rzehak H, Weiglein G (2010). Nucl. Phys. Proc. Suppl..

[98] Peccei RD, Quinn HR (1977). Phys. Rev. Lett..

[99] Dimopoulos S, Thomas SD (1996). Nucl. Phys. B.

[100] Fuchs E, Weiglein G (2017). JHEP.

[101] Küblbeck J, Böhm M, Denner A (1990). Comput. Phys. Commun..

[102] Hahn T (2001). Comput. Phys. Commun..

[103] A. von Manteuffel, C. Studerus, (2012). arXiv:1201.4330

[104] Hahn T, Pérez-Victoria M (1999). Comput. Phys. Commun..

[105] Freitas A, Stockinger D (2002). Phys. Rev. D.

[106] Sperling M, Stöckinger D, Voigt A (2013). JHEP.

[107] Sperling M, Stöckinger D, Voigt A (2014). JHEP.

[108] Baro N, Boudjema F, Semenov A (2008). Phys. Rev. D.

[109] Heinemeyer S, Rzehak H, Schappacher C (2010). Phys. Rev. D.

[110] Banks T (1988). Nucl. Phys. B.

[111] Hall L, Rattazzi R, Sarid U (1994). Phys. Rev. D.

[112] Hempfling R (1994). Phys. Rev. D.

[113] Carena M, Olechowski M, Pokorski S, Wagner C (1994). Nucl. Phys. B.

[114] Carena M, Garcia D, Nierste U, Wagner C (2000). Nucl. Phys. B.

[115] Eberl H, Hidaka K, Kraml S, Majerotto W, Yamada Y (2000). Phys. Rev. D.

[116] Takagi T (1927). Jpn. J. Math..

[117] Bloch S, Vanhove P (2015). J. Number Theory.

[118] Bloch S, Kerr M, Vanhove P (2015). Compos. Math..

[119] Adams L, Bogner C, Weinzierl S (2013). J. Math. Phys..

[120] Remiddi E, Tancredi L (2014). Nucl. Phys. B.

[121] Adams L, Bogner C, Weinzierl S (2015). J. Math. Phys..

[122] Adams L, Bogner C, Weinzierl S (2016). J. Math. Phys..

[123] S. Bloch, M. Kerr, P. Vanhove, (2016). arXiv:1601.08181

[124] Remiddi E, Tancredi L (2016). Nucl. Phys. B.

[125] Adams L, Bogner C, Schweitzer A, Weinzierl S (2016). J. Math. Phys..

[126] J. Brödel, C. Duhr, F. Dulat, L. Tancredi, (2017). arXiv:1712.07095

[127] Agrawal S, Hahn T, Mirabella E (2012). J. Phys. Conf. Ser..

[128] Hahn T (2005). Comput. Phys. Commun..

[129] Carena M, Heinemeyer S, Stål O, Wagner CEM, Weiglein G (2013). Eur. Phys. J. C.

[130] Arbey A, Ellis J, Godbole R, Mahmoudi F (2015). Eur. Phys. J. C.

[131] Bagnaschi E, Harlander R, Liebler S, Mantler H, Slavich P, Vicini A (2014). JHEP.

[132] Frere J, Jones T, Raby S (1983). Nucl. Phys. B.

[133] Gunion J, Haber H, Sher M (1988). Nucl. Phys. B.

[134] Casas J, Lleyda A, Muñoz C (1996). Nucl. Phys. B.

[135] Hisano J, Nagai M, Paradisi P (2006). Phys. Lett. B.

[136] Hisano J, Nagai M, Paradisi P (2008). Phys. Rev. D.

[137] Hisano J, Nagai M, Paradisi P (2009). Phys. Rev. D.

[138] Camargo-Molina J, O’Leary B, Porod W, Staub F (2013). Eur. Phys. J. C.

[139] Hollik WG (2016). JHEP.

[140] Collaboration CMS (2014). JHEP.

[141] ATLAS Collaboration, (2016). CDS 2206278

